# A Computational Workflow Integrating Explainable AI and Database Mining for the Prioritization of Antimicrobial Natural Products

**DOI:** 10.3390/molecules31203683

**Published:** 2026-10-14

**Authors:** Pietro Hiram Guzzi, Annamaria Defilippo, Pierangelo Veltri, Giancarlo Ceccarelli, Fabio Scarpa, Massimo Ciccozzi, Francesco Branda

**Affiliations:** 1 Department of Surgical and Medical Sciences, Magna Graecia University of Catanzaro, Viale Europa, 88100 Catanzaro, Italy; hguzzi@unicz.it (P.H.G.); annamaria.defilippo@studenti.unicz.it (A.D.); 2 Department of Computer Engineering, Modeling, Electronics and Systems (DIMES), University of Calabria, Via P. Bucci, 87036 Rende, Italy; pierangelo.veltri@unical.it; 3 Department of Public Health and Infectious Diseases, University Hospital Policlinico Umberto I, Sapienza University of Rome, 00185 Rome, Italy; giancarlo.ceccarelli@uniroma1.it; 4 Department of Biomedical Sciences, University of Sassari, 07100 Sassari, Italy; fscarpa@uniss.it; 5 Unit of Medical Statistics and Molecular Epidemiology, Università Campus Bio-Medico di Roma, 00128 Rome, Italy; m.ciccozzi@unicampus.it

**Keywords:** antimicrobial resistance, natural products, machine learning, explainable artificial intelligence, conformal prediction, uncertainty quantification, multi-objective optimization, scaffold splitting, virtual screening, antibiotic discovery

## Abstract

The escalating threat of antimicrobial resistance demands the discovery of antibacterial agents with unprecedented chemical scaffolds. Natural products have historically been the most prolific source of antibiotics, yet the vast majority of catalogued natural products remain untested for antimicrobial activity. Here we present Natural-Product Graph–language, Uncertainty-Aware Relational Discovery (NP-GUARD), a modular computational framework that reframes antimicrobial natural-product discovery as an uncertainty-aware, relational, multi-objective learning problem. NP-GUARD integrates six core components: provenance-tracked data curation, multi-view molecular representation, pathogen-conditioned multi-task prediction, explicit uncertainty quantification, stability-aware explainability, and conformal-Pareto prioritization, all consolidated within a queryable knowledge base. Of these components, the present study implements and benchmarks the data-curation, predictive, uncertainty, explainability and prioritization layers, including a first pathogen-conditioned masked multi-task graph model, while the full multi-view encoder and the knowledge base are specified for subsequent work. We report a proof-of-concept evaluation on public screening data for *Staphylococcus aureus* (38,995 compounds), *Escherichia coli* (2291) and *Pseudomonas aeruginosa* (29,986), together with human cytotoxicity data and a 406,959-compound screening library from COCONUT. Under repeated scaffold-aware splitting, baseline models achieve strong discrimination (AUROC 0.78–0.93) but reveal substantial split-to-split variance in minority-class performance; class-conditional conformal prediction attains near-nominal per-class coverage; and feature attributions show only moderate stability across independently trained models (top-20 Jaccard 0.54), underscoring a critical caveat for explanation-driven discovery. Screening the COCONUT library validates the uncertainty-aware activity ranking, achieving an 87-fold enrichment of known antibiotics in the top 100 candidates (128-fold within the fully characterized named-compound stratum), although analogue-exclusion analysis shows this signal is dominated by rediscovery of training-like chemistry. The novelty-seeking Pareto layer yields a structurally diverse 90-compound shortlist spanning 72 scaffolds, deliberately steered toward unexplored chemotypes; we emphasize that the shortlist constitutes prioritized hypotheses for experimental testing, not validated actives. NP-GUARD provides a transparent, reproducible and decision-oriented foundation for prioritizing natural products for experimental validation.

## 1. Introduction

Antimicrobial resistance (AMR) is among the most pressing threats to global health, with the World Health Organization declaring it one of the top ten global public-health challenges. In 2019, bacterial AMR was directly responsible for an estimated 1.27 million deaths and associated with 4.95 million deaths worldwide, a burden that is projected to escalate substantially over the coming decades [1,2]. The clinical pipeline of new antibiotics, however, remains alarmingly sparse: most drugs approved in recent years are derivatives of established scaffolds, and genuinely new structural classes active against priority Gram-negative pathogens are exceedingly rare [3,4]. The discovery of antimicrobial agents with novel chemotypes is therefore a central scientific and public-health imperative.

Natural products (NPs) have historically been the single most productive source of antibacterial scaffolds, accounting for a large fraction of approved antibiotics either as native compounds, semi-synthetic derivatives, or synthetic molecules inspired by natural pharmacophores [5,6]. NPs occupy regions of chemical space that are distinct from conventional synthetic screening libraries: they exhibit greater scaffold complexity, higher fractions of $sp^{3}$-hybridized carbons, and richer stereochemistry, properties that have been associated with the ability to modulate challenging biological targets, including bacterial protein–protein interactions and membrane-bound enzymes [7,8]. Over the past decade, public natural-product resources have expanded dramatically: COCONUT, LOTUS, NPASS, and the Natural Products Atlas now collectively index well over one million NP structures with varying degrees of structural and bioactivity annotation [9,10,11,12]. Yet systematic experimental screening of these vast collections against microbial pathogens remains prohibitively expensive, time-consuming, and low-throughput; as a consequence, the antimicrobial activity of the overwhelming majority of catalogued natural products remains completely unknown.

Machine learning (ML) has emerged as a powerful complement to experimental screening, offering the potential to prioritize compounds for testing from enormous chemical libraries. Deep-learning models trained on measured growth-inhibition data have already demonstrated the ability to identify antibiotics that are structurally distant from known agents. Stokes and colleagues discovered halicin, a broad-spectrum antibiotic with a novel mechanism, using a directed message-passing neural network trained on a relatively small dataset of 2335 compounds [13]. Liu and colleagues applied a similar approach to discover abaucin, a narrow-spectrum agent against *Acinetobacter baumannii*, by designing a focused screening strategy that leveraged deep-learning predictions [14]. More recently, Wong and colleagues identified new structural classes of antibiotics active against methicillin-resistant *Staphylococcus aureus* through explainable graph neural networks that highlighted substructural rationales [15]. These successes underscore the capacity of learned molecular representations, whether derived from graph convolutional networks, SMILES-based language models, or hybrid architectures, to generalize beyond the chemical series present in the training data [16,17,18,19,20]. In parallel, the broader biomedical research community has increasingly adopted integrative approaches that combine diverse data types, from clinical biomarkers and proteomic interaction networks to graph-based patient-triage systems, demonstrating the value of relational and multi-modal modelling in complex biological contexts [21,22,23]. Yet, despite these advances, several critical methodological gaps continue to constrain the translation of computational screening into actionable experimental programmes.

First, the majority of published models are trained and evaluated on a single pathogen and a single activity endpoint, even though antimicrobial utility is inherently multi-dimensional: it depends jointly on potency against a panel of relevant pathogens, absence of mammalian cytotoxicity, favourable pharmacokinetic and ADMET properties, and low propensity for resistance development. A compound that is highly active against *S. aureus* but toxic to human cells, or one that is active in vitro but poorly soluble, is unlikely to become a viable therapeutic lead [24]. Multi-task and multi-objective approaches are increasingly advocated to address this complexity, yet they remain underutilised in antimicrobial discovery [25]. Second, predictive uncertainty is rarely quantified in antimicrobial ML studies, and even more seldom propagated into the candidate-ranking decision. A molecule receiving an extreme but highly uncertain activity score is often treated equivalently to one with a robustly supported prediction, leading to inefficient allocation of experimental resources [26]. Conformal prediction and deep ensembles have recently been proposed as principled frameworks for uncertainty-aware molecular property prediction, but their adoption in antimicrobial screening is still nascent [27,28]. Third, random train–test splitting of chemical data is known to severely overestimate generalization to novel scaffolds, because near-identical analogues frequently appear in both training and test sets [29,30]. Scaffold-aware splitting, which separates compounds by their Bemis–Murcko core frameworks, provides a more realistic assessment of a model’s ability to discover genuinely new chemotypes [31,32]. Fourth, interpretability analyses are typically reported for a single fitted model without assessing whether the highlighted chemical features are stable across independently trained models, a prerequisite for trusting explanations as mechanistic hypotheses. Recent work has shown that post-hoc attributions can be highly sensitive to model initialization and training data, raising concerns about their reliability for guiding compound optimization [33]. Finally, natural products are severely underrepresented in the compound collections used to train most published models, which are often dominated by synthetic screening libraries. As a result, the chemical space in which NPs are most promising, characterised by distinctive stereochemical and structural features, is precisely the space in which models are least reliable, leading to a critical domain-shift problem [19].

To address these challenges, we introduce Natural-Product Graph–language, Uncertainty-Aware Relational Discovery (NP-GUARD), a modular computational framework that reframes antimicrobial natural-product discovery as an uncertainty-aware, relational, and multi-objective learning problem. NP-GUARD integrates six core components: (i) versioned, provenance-tracked curation of heterogeneous natural-product and antimicrobial-assay resources; (ii) natural-product-aware multi-view molecular representation that combines graph, chemical-language, and interpretable-concept views with cross-modal fusion and domain adaptation; (iii) pathogen-conditioned multi-task prediction of antimicrobial activity together with ADMET and toxicity endpoints; (iv) explicit uncertainty quantification, calibration, and applicability-domain estimation; (v) multi-resolution explainability with formal stability analysis; and (vi) conformal-Pareto multi-objective prioritisation with diversity-aware candidate selection, all consolidated in a queryable knowledge base. This design explicitly separates representation, prediction, trustworthiness, and decision-making, enabling the system to recommend compounds not solely on the basis of high predicted activity, but also on their reliability, novelty, and structural diversity.

In this paper, we describe the NP-GUARD architecture in full and report a proof-of-concept evaluation of its data-curation, predictive, uncertainty, explainability, and prioritisation components using public antimicrobial screening data for three priority pathogens, i.e., *S. aureus*, *Escherichia coli*, and *Pseudomonas aeruginosa*, together with human cytotoxicity data and a screening library of more than 400,000 natural products from COCONUT. Employing a leakage-resistant, repeated scaffold-aware evaluation protocol, we demonstrate that baseline models instantiated within the framework achieve competitive discrimination while providing calibrated, class-conditional prediction sets with near-nominal coverage. We further quantify the moderate stability of model explanations across independently trained models, highlighting a previously underappreciated caveat for explanation-guided discovery. Finally, we show that uncertainty-adjusted multi-objective ranking recovers a structurally diverse candidate set enriched for known antibiotics from the natural-product library, while deliberately steering toward novel chemotypes. The full deep architecture, including the cross-modal encoder and pathogen-conditioned mixture-of-experts model, is specified here and benchmarked in subsequent work.

## 2. Background

### 2.1. Machine Learning for Antibiotic Discovery

The application of machine learning to antibiotic discovery has undergone a fundamental transformation over the past decade, evolving from classical quantitative structure–activity relationship (QSAR) models built on hand-crafted molecular descriptors and fingerprints to sophisticated deep-learning architectures that learn task-specific representations directly from molecular graphs or SMILES strings. This evolution has been driven by the recognition that traditional computational approaches, while valuable for exploring well-defined chemical series, often fail to generalize to structurally novel compounds, precisely the chemotypes most urgently needed to address the growing crisis of antimicrobial resistance. The limitations of conventional approaches are particularly acute in the context of natural-product discovery, where the chemical space is vast, structurally complex, and poorly represented in typical screening libraries, creating a fundamental challenge for computational methods that rely on similarity to known active compounds.

A landmark study by Stokes and colleagues demonstrated the power of deep learning for antibiotic discovery by training a directed message-passing neural network on growth-inhibition measurements for 2335 compounds against Escherichia coli and prospectively identifying halicin, a broad-spectrum antibiotic with a novel mechanism of action that was structurally distant from known antibacterial agents [13]. The study employed a rigorous experimental validation pipeline, demonstrating that halicin was effective against a range of multidrug-resistant pathogens, including *Acinetobacter baumannii*, *Klebsiella pneumoniae*, and *Enterobacteriaceae*, and exhibited low toxicity to human cells. The discovery of halicin was particularly notable because the compound was originally developed as a potential treatment for diabetes and had never been considered for antimicrobial applications, illustrating the capacity of deep learning to identify unexpected therapeutic applications for known compounds. This work established that deep neural networks could learn molecular representations capable of generalizing beyond the chemical series present in the training data, a finding that has profoundly influenced subsequent research in the field and provided a proof-of-concept for the use of artificial intelligence in antibiotic discovery. The study also highlighted the importance of using diverse training data, as the model’s ability to identify structurally novel compounds was attributed to the broad chemical diversity of the training set.

Building on this paradigm, Liu and colleagues combined a similar architecture with a narrow-spectrum screening design to discover abaucin, a selective agent against the multidrug-resistant pathogen Acinetobacter baumannii [14]. Unlike halicin, which exhibited broad-spectrum activity, abaucin was specifically designed to target *A. baumannii*, a pathogen of critical priority due to its high levels of drug resistance and the limited treatment options available. The study employed a focused screening strategy that leveraged deep-learning predictions to select compounds from a diverse chemical library, followed by experimental validation of the top candidates. The researchers screened over 7500 compounds and identified abaucin as a potent and selective inhibitor of *A. baumannii* growth, with minimal activity against other bacterial species. Mechanistic studies revealed that abaucin disrupts the function of the lipoprotein trafficking pathway, a novel mechanism of action that differs from conventional antibiotics and holds promise for overcoming existing resistance mechanisms. This work demonstrated that deep learning models could be effectively directed toward specific, clinically relevant targets through careful screening design, highlighting the versatility of the approach and its potential for addressing the challenge of pathogen-specific antibiotic discovery. The study also underscored the importance of narrow-spectrum antibiotics as a strategy for minimizing collateral damage to the microbiome and reducing the selective pressure that drives resistance development.

More recently, Wong and colleagues conducted a large-scale screen of 39,312 compounds against *Staphylococcus aureus* with parallel human cytotoxicity measurements, trained ensembles of graph neural networks, and used explainable graph algorithms to extract substructural rationales that defined new antibiotic classes [15]. This study represents a significant advance in the integration of prediction, explainability, and experimental validation, demonstrating that model-derived molecular rationales can guide the discovery of structurally distinct antibiotic classes. The researchers identified a structural class of antibiotics with activity against methicillin-resistant *S. aureus* (MRSA) and validated the findings through a series of experimental assays, including minimum inhibitory concentration (MIC) determinations, cytotoxicity profiling, and in vivo efficacy studies in mouse models of infection. The study also demonstrated that the identified compounds were effective against MRSA strains resistant to multiple existing antibiotics, highlighting the potential of deep learning to discover compounds that circumvent existing resistance mechanisms. The three studies collectively establish both the feasibility of deep-learning-guided antibiotic discovery and the importance of coupling activity prediction with cytotoxicity assessment, a design principle that NP-GUARD adopts explicitly and extends through its multi-objective and uncertainty-aware prioritization framework.

### 2.2. Molecular Representation Learning

Molecular representations used in property prediction span four broad families, each with distinct strengths and limitations that influence their applicability to antimicrobial discovery. The first family comprises fixed descriptors and structural fingerprints, including Morgan/circular fingerprints and physicochemical property vectors. These representations remain strong baselines when paired with ensemble tree methods, as they are computationally efficient, chemically interpretable, and well-established in the cheminformatics literature [29]. The Extended Connectivity Fingerprint (ECFP), also known as Morgan fingerprint, is particularly widely used due to its ability to capture circular substructures around each atom, providing a rich representation of molecular topology. The ECFP algorithm generates fingerprints by iteratively updating atom identifiers based on the identifiers of neighboring atoms and bonds, creating a representation that captures structural features at multiple radii. This approach has been extensively validated in drug discovery applications and remains a benchmark for molecular similarity searching and property prediction. Physicochemical property vectors, which include features such as molecular weight, logP, polar surface area, and hydrogen-bond donor/acceptor counts, complement fingerprint-based representations by capturing global molecular properties that influence drug-likeness and bioavailability. These properties are derived from fundamental chemical principles and provide interpretable features that can be related to biological activity through established structure–activity relationships. The primary limitation of fixed descriptors is that they are determined at the time of feature engineering and cannot adapt to the specific properties of the prediction task, potentially missing subtle structural patterns that are important for antimicrobial activity.

The second family consists of graph neural networks (GNNs), notably the directed message-passing neural network (D-MPNN) implemented in Chemprop [16,34]. These architectures learn representations from atom and bond features through iterative message-passing operations that capture local chemical environments and propagate information across the molecular graph. D-MPNNs currently define the state of the art for many property-prediction tasks, as they can learn task-specific representations directly from molecular structure without requiring hand-crafted features. The message-passing paradigm, originally inspired by the Weisfeiler–Lehman graph isomorphism test, enables the network to capture increasingly complex structural patterns as information propagates through the graph. In a D-MPNN, messages are passed along directed edges, allowing the model to distinguish between different bond directions and capture the directional nature of chemical bonds. This directional information is particularly important for capturing stereochemical relationships and the orientation of functional groups, which can significantly influence biological activity. Their effectiveness has been demonstrated across a wide range of chemical property prediction benchmarks, including antimicrobial activity, and they form the backbone of several recent antibiotic discovery studies [14,15,34]. The D-MPNN architecture has been further extended through the incorporation of bond-directionality, which allows the model to distinguish between different bond orders and stereochemical configurations, providing a more nuanced representation of molecular structure. Recent variants have also incorporated attention mechanisms and graph transformer layers, enabling the model to capture long-range interactions and global molecular features that are not captured by local message passing alone.

The third family comprises chemical language models pretrained on large SMILES corpora with masked-token objectives. Models such as ChemBERTa and MolFormer provide transferable sequence-based representations that capture chemical and structural information from unlabeled molecular sequences [17,20,35]. These approaches leverage the vast quantities of available chemical data and have demonstrated strong performance on downstream molecular property prediction tasks, particularly when labelled data are limited. The underlying principle is that pretraining on a large corpus of chemical structures allows the model to learn general features of molecular chemistry that can be fine-tuned for specific prediction tasks. MolFormer, for instance, was trained on a dataset of over 1.1 billion SMILES strings, making it one of the largest chemical language models to date and demonstrating that scale is a critical factor in learning transferable molecular representations [17]. The success of these models has parallels in natural language processing, where large-scale pretraining has revolutionized the field, and suggests that similar advances are possible in cheminformatics. Chemical language models operate by treating SMILES strings as sequences of tokens and applying transformer architectures to learn contextual representations of each token. The attention mechanism in transformers allows the model to capture long-range dependencies and interactions between distant parts of the molecule, which is particularly important for understanding the three-dimensional structure and conformational flexibility of complex molecules. However, the sequential nature of SMILES representations can be a limitation, as different SMILES strings can represent the same molecule (non-canonical SMILES), requiring careful preprocessing to ensure consistent representation.

The fourth family encompasses self-supervised graph pretraining schemes such as GROVER and geometry-aware models such as Uni-Mol, which further enrich molecular encoders with large unlabeled corpora and three-dimensional information [18,36]. These approaches integrate multiple sources of molecular information, including two-dimensional topology, three-dimensional conformation, and biochemical properties, to create rich representations that capture the complexity of molecular structure and function. GROVER, for example, employs a graph-transformer architecture with self-supervised pretraining objectives that include atom-level and bond-level masked prediction tasks, learning representations that capture both local and global molecular features [36]. The graph-transformer architecture combines the local feature extraction capabilities of graph neural networks with the global attention mechanisms of transformers, enabling the model to capture both fine-grained structural details and broader molecular context. Uni-Mol extends this approach by incorporating three-dimensional molecular conformations, which are particularly important for understanding molecular interactions with biological targets [18]. The 3D conformations are generated using molecular dynamics simulations or conformation generation algorithms, and the model learns to predict properties that depend on the three-dimensional structure of the molecule, such as binding affinity and conformational stability. The integration of 3D information is especially relevant for natural products, which often exhibit complex stereochemistry that influences their biological activity. The challenge with 3D methods is the computational cost of generating conformations and the uncertainty associated with predicted conformations, which can propagate into the model predictions.

Recent work on pretrained molecular representations, exemplified by MolE, shows that representations learned from unlabeled structures transfer to antimicrobial-activity prediction even when labelled data are scarce [19]. This finding is particularly relevant for natural-product discovery, where labelled antimicrobial activity data are limited relative to the vast chemical space of natural products. The study demonstrated that MolE representations consistently outperform traditional fingerprints and other pretrained representations across multiple antimicrobial datasets, highlighting the value of large-scale pretraining for this application domain. The study also showed that the representations captured meaningful chemical features related to antimicrobial activity, suggesting that the pretraining objectives were aligned with the downstream task. However, natural products differ systematically from the synthetic compounds that dominate pretraining corpora, exhibiting greater stereochemical complexity, higher fractions of sp^3^-hybridized carbons, and more diverse ring systems [7,8]. This domain shift motivates the natural-product-specific adaptation and multi-view fusion strategies employed in NP-GUARD, which are designed to bridge the gap between generic molecular representations and the distinctive chemistry of natural products. The domain adaptation component of NP-GUARD uses a large corpus of unlabeled natural products to refine the pretrained representations, aligning them with the distribution of natural-product chemistry before supervised training on antimicrobial activity data.

### 2.3. Uncertainty Quantification and Conformal Prediction

Trustworthy prioritization of compounds for experimental testing requires knowing when a prediction should not be trusted. In antimicrobial discovery, where experimental resources are limited and false positives can lead to costly validation efforts, the ability to distinguish confident predictions from uncertain ones is essential for efficient resource allocation. Despite its importance, predictive uncertainty is rarely quantified in antimicrobial machine-learning studies, and even more seldom propagated into the candidate-ranking decision, a critical gap that NP-GUARD addresses explicitly. The consequences of ignoring uncertainty are significant: a molecule receiving an extreme but highly uncertain activity score is often treated equivalently to one with a robustly supported prediction, leading to inefficient allocation of experimental resources and potentially missed opportunities for discovery. The problem is particularly acute in antimicrobial discovery because the chemical space is vast and the training data are often sparse, leading to regions of the space where predictions are inherently uncertain.

Deep ensembles remain a strong and simple baseline for predictive uncertainty in molecular models [28]. By training multiple independently initialized models and aggregating their predictions, deep ensembles provide an empirical measure of epistemic variability that captures the uncertainty arising from different model initializations and training trajectories. For regression endpoints, the ensemble mean and variance provide point estimates and uncertainty intervals; for classification, the ensemble probabilities reflect the diversity of model predictions. Deep ensembles have been widely adopted in molecular property prediction and provide a robust foundation for uncertainty estimation. The approach has been shown to outperform more complex Bayesian methods in many practical settings, while being computationally simpler to implement. In the context of antimicrobial discovery, deep ensembles have been used to identify compounds with high predicted activity and low prediction variance, focusing experimental validation on the most reliable candidates. The diversity of the ensemble members is crucial for effective uncertainty estimation; if the models are too similar, the ensemble will underestimate uncertainty. This can be addressed through the use of different random seeds, different training data subsets, or different model architectures.

Conformal prediction provides distribution-free, finite-sample coverage guarantees by wrapping any point predictor in a calibration procedure [27,37]. Unlike Bayesian or ensemble methods that provide approximate uncertainty estimates, conformal prediction offers rigorous statistical guarantees: for a specified miscoverage level α, the prediction sets will contain the true label with probability at least 1−α, without requiring assumptions about the underlying data distribution. This property has recently been advocated for molecular property prediction and drug-discovery applications, as it enables reliable decision-making under uncertainty [26]. The calibration procedure requires a held-out dataset, which is used to compute nonconformity scores that measure how unusual a new prediction is relative to the calibration examples. The resulting prediction sets have a clear interpretation: for a given molecule, the set contains all labels that are sufficiently consistent with the calibration data. The finite-sample guarantee is a powerful property, as it provides a statistical basis for decision-making that is not available with other uncertainty quantification methods.

Importantly, conformal prediction can be applied to any underlying model, making it a flexible complement to deep ensembles and other uncertainty quantification methods. The combination of deep ensembles for uncertainty estimation and conformal prediction for coverage guarantees provides a powerful framework for trustworthy molecular property prediction. Because antimicrobial datasets are typically highly imbalanced, with active compounds often representing less than 5% of screened libraries, marginal coverage can hide poor coverage of the active class. NP-GUARD therefore employs class-conditional (Mondrian) conformal prediction, which guarantees coverage separately for actives and inactives. This ensures that the active class, the class of primary interest in antimicrobial discovery, receives the same coverage guarantees as the majority inactive class, preventing the model from providing overly confident predictions for the rare but critical active compounds. The class-conditional approach is particularly important in the context of antimicrobial discovery, where the active class is of primary interest but is also the class with the smallest sample size, making it the most difficult to calibrate reliably. The finite-sample limits of conformal prediction on very small minority classes are an important consideration, as the method requires sufficient calibration data for each class to estimate reliable quantiles.

Applicability-domain estimation complements these techniques by identifying candidates that lie outside the chemical space represented in the training set. For compounds that are structurally or representationally distant from the training data, no reliable prediction can be expected, regardless of the model’s confidence. NP-GUARD integrates applicability-domain estimation into its prioritization framework, so that out-of-domain compounds are flagged for experimental caution rather than discarded outright, an important consideration for natural-product discovery, where structural novelty is explicitly sought. The applicability-domain score can be computed using a variety of approaches, including distance-based methods in fingerprint or learned-representation space, density estimation, or ensemble-based measures of model agreement. By combining uncertainty quantification, conformal prediction, and applicability-domain estimation, NP-GUARD provides a comprehensive framework for trustworthy prediction that supports informed decision-making in antimicrobial natural-product discovery. The framework ensures that candidates are evaluated not only on their predicted activity but also on the reliability of those predictions, providing a more robust basis for experimental prioritization.

### 2.4. Explainable Artificial Intelligence in Chemistry

Post-hoc explanation methods attribute model predictions to input features, providing insights into the chemical rationales underlying a model’s decisions. These explanations are valuable for hypothesis generation, model debugging, and building trust in computational predictions. Several explanation methods have been applied in the molecular sciences, each with distinct strengths and limitations. The interpretability of machine learning models is particularly important in drug discovery, where understanding the chemical features that drive activity can guide compound optimization and suggest new directions for synthetic chemistry. In the context of antimicrobial discovery, explanations can help researchers understand why a particular compound is predicted to be active, enabling them to focus on the most promising chemical features in subsequent screening campaigns.

SHapley Additive exPlanations (SHAP) values provide additive feature attributions grounded in cooperative game theory, offering a principled framework for quantifying the contribution of each feature to a model’s prediction [38]. For molecular models, SHAP can attribute predictions to individual molecular descriptors or fingerprint bits, enabling identification of the physicochemical properties that drive antimicrobial activity. The SHAP framework builds on Shapley values from cooperative game theory, which allocate the contribution of each feature by considering all possible subsets of features. This approach has the desirable property of consistency: if a model changes so that it depends more on a particular feature, the SHAP value for that feature will not decrease. However, computing exact Shapley values is computationally expensive, and the tree-specific implementation (TreeSHAP) is typically used for ensemble models [39]. TreeSHAP exploits the structure of decision trees to compute Shapley values efficiently, making it practical for large models and large datasets. The resulting attributions are consistent with the model’s predictions and provide a global view of feature importance across the entire dataset.

Integrated gradients attribute predictions of differentiable models along a baseline path, providing a method for attributing predictions to input features that is consistent with the model’s architecture [40]. The method satisfies several desirable axioms, including sensitivity and implementation invariance, making it a principled approach for attribution in deep learning models. For molecular graph models, integrated gradients can be applied to atom and bond features, identifying the substructures most influential for a prediction. However, the choice of baseline can significantly affect the resulting attributions, and multiple baseline choices may be necessary to obtain robust explanations. The baseline is typically a reference point that represents the absence of features, such as a zero vector or a reference molecule, and the attributions are computed along a path from the baseline to the input. The choice of baseline is particularly important in chemistry, where the absence of a feature may not have a meaningful chemical interpretation.

GNNExplainer identifies compact subgraphs responsible for graph-neural-network predictions, generating chemically interpretable rationales in the form of molecular substructures [41]. The method optimizes a mask over edges and node features to find the smallest subgraph that is most influential for the prediction, providing a visual and chemically interpretable explanation. In the antimicrobial context, substructure-level explanations have been used to define candidate antibiotic classes [15]. By identifying the molecular substructures most influential for predicted activity, explainability methods can guide compound optimization and suggest chemical features to prioritize in subsequent screening efforts. The subgraph explanations are particularly appealing because they correspond to chemically meaningful units, such as functional groups or ring systems, that can be synthesized and tested experimentally. The method also provides a measure of the importance of each atom and bond, enabling a detailed understanding of the structural features that drive the prediction.

However, a critical but rarely tested assumption is that explanations are reproducible: if independently trained models highlight different substructures, explanation-guided optimization may chase noise rather than robust chemical principles. Recent work has shown that post-hoc attributions can be highly sensitive to model initialization and training data, raising concerns about their reliability for guiding compound optimization [33]. The instability of explanations is particularly problematic when explanations are used to guide chemical optimization, as small changes in the model can lead to different recommendations. NP-GUARD therefore treats explanation stability across resampled model fits as a first-class evaluation quantity. By calculating explanations across ensemble members and resampling folds, the framework quantifies the reproducibility of feature attributions and restricts explanation-guided optimization to features that demonstrate sufficient recurrence across independently fitted models. This approach converts explainable AI from a purely illustrative component into an experimentally assessable property of the computational framework, providing a more rigorous foundation for explanation-driven discovery. Only explanations that demonstrate sufficient stability are treated as robust mechanistic hypotheses, ensuring that optimization efforts are directed toward the most reliable chemical features.

### 2.5. Multi-Objective Prioritization and Chemical Diversity

Prioritizing compounds for experimental testing is intrinsically multi-objective: antimicrobial potency must be balanced against toxicity, pharmacokinetic suitability, novelty, and structural diversity. A compound that is highly active against a target pathogen but toxic to human cells, or one that is active in vitro but poorly soluble, is unlikely to become a viable therapeutic lead. Similarly, a screening campaign that returns twenty close analogues of the same scaffold provides considerably less discovery value than one returning a chemically diverse set of high-quality candidates. The multi-objective nature of drug discovery has been recognized for decades, but the integration of multiple objectives in computational screening remains challenging due to the difficulty of specifying appropriate trade-offs between competing goals. Traditional approaches that combine objectives into a single composite score require arbitrary weighting of the objectives, which can lead to suboptimal selections if the weights are not well-aligned with the decision-maker’s preferences. Furthermore, the relative importance of different objectives may vary depending on the application context, making it difficult to define a single objective function that is universally applicable.

Pareto optimality provides a principled notion of non-dominated trade-offs: a molecule belongs to the Pareto set if no alternative candidate is at least as favorable for every objective and strictly superior for at least one. This concept, derived from welfare economics, has been widely applied in multi-objective optimization. In the context of molecular discovery, the Pareto front represents the set of compounds that represent optimal compromises between competing objectives. Evolutionary multi-objective algorithms such as NSGA-II are widely used to approximate Pareto fronts in molecular discovery and drug design [42]. These approaches enable systematic exploration of trade-offs between competing objectives, identifying molecules that represent optimal compromises rather than extreme optima on a single objective. NSGA-II employs a fast non-dominated sorting procedure and a crowding distance mechanism to maintain diversity in the population, ensuring that the algorithm explores a wide range of the Pareto front. The crowding distance measures how close each solution is to its neighbors, promoting the selection of diverse solutions that are spread across the Pareto front. This diversity is particularly important in molecular discovery, where the goal is to identify a diverse set of candidate compounds rather than a single optimal compound.

In molecular discovery, diversity-aware selection avoids shortlists dominated by a single scaffold family. Clustering candidates in fingerprint or learned-representation space enables selection of diverse compounds that sample different regions of chemical space, increasing the probability of discovering novel chemotypes. The diversity of a candidate set can be quantified using measures such as the average pairwise similarity or the number of unique Bemis–Murcko scaffolds [31]. Bemis–Murcko scaffolds represent the core molecular framework of a compound, excluding side chains and functional groups, and provide a useful abstraction for assessing scaffold diversity. NP-GUARD combines these principles with conformal uncertainty bounds, so that a candidate is favored only when the evidence supporting its activity is sufficiently reliable, a strategy termed conformal-Pareto prioritization.

This approach distinguishes NP-GUARD from conventional virtual-screening pipelines that rank compounds by a single composite score. By explicitly modelling uncertainty, novelty, and diversity alongside antimicrobial activity, the framework provides a more principled basis for experimental prioritization, directing resources toward compounds that are not only predicted to be active but also represent novel, diverse, and reliable candidates for further investigation. The conformal-Pareto approach explicitly incorporates uncertainty into the decision-making process: for beneficial endpoints, the lower conformal confidence bound is used, while for undesirable endpoints such as toxicity, the upper confidence bound is applied. This conservative approach ensures that a compound does not become Pareto-optimal merely because it has an extreme but highly uncertain point prediction. The integration of uncertainty into multi-objective optimization is a distinctive feature of NP-GUARD, providing a more robust basis for decision-making under uncertainty. The framework also supports interactive exploration of the Pareto front, allowing users to adjust the relative importance of different objectives and explore the trade-offs between them.

### 2.6. Natural-Product and Antimicrobial Data Resources

The data foundation of NP-GUARD builds on open natural-product collections, i.e., COCONUT [9], LOTUS [10], NPASS [11], and the Natural Products Atlas [12], and on antimicrobial activity resources including CO-ADD [43], ChEMBL [44], and PubChem BioAssay [45]. These resources differ in identifier schemes, structure conventions, assay endpoints, and units, making integration and harmonization a substantial computational task. The heterogeneity of these resources reflects the diversity of sources from which they are derived, including the scientific literature, high-throughput screening campaigns, and individual research contributions. The integration of these resources requires careful attention to data provenance and quality control to ensure that the resulting dataset is reliable and reproducible.

COCONUT (Collection of Open Natural Products) is one of the largest publicly available natural-product databases, providing structure, source organism, and bioactivity information for over 700,000 compounds [9]. The database is curated from a wide range of sources, including the scientific literature and other public databases, and includes compounds from all kingdoms of life. The current release, COCONUT 2.0, represents a significant update to the database, with improved curation, enhanced metadata, and expanded coverage of natural-product chemistry. The database includes information on compound structures, source organisms, and biological activities, providing a comprehensive resource for natural-product discovery. The structures are stored as SMILES strings and InChI keys, enabling efficient searching and comparison of compounds. The database also includes links to external resources such as PubMed and the Chemical Abstracts Service, facilitating the retrieval of additional information about individual compounds.

LOTUS extends this foundation with a comprehensive natural-products knowledge graph that integrates structural, taxonomic, and bibliographic information [10]. The LOTUS database is notable for its rigorous curation and its integration of data from multiple sources, providing a unified view of natural-product chemistry. The knowledge graph structure enables queries that combine different types of information, such as compounds from a particular organism with known biological activity. The database includes detailed information on source organisms, including taxonomic classification and geographical distribution, enabling researchers to explore the relationship between compound structure and biological source. The bibliographic information provides links to the original literature, enabling researchers to trace the provenance of each compound and activity measurement.

NPASS (Natural Product Activity and Species Source Database) provides detailed bioactivity data, including quantitative measurements against a wide range of biological targets [11]. The database includes information on compound activity, species source, and reference information, making it a valuable resource for understanding the relationship between natural-product structure and biological function. The NPASS database is updated regularly and includes data from both the scientific literature and high-throughput screening campaigns. The quantitative activity data, including IC50 and MIC values, provide a foundation for building predictive models of antimicrobial activity.

The Natural Products Atlas curates microbially derived natural products with detailed provenance information [12]. The database focuses specifically on compounds produced by bacteria and fungi, which are among the most prolific sources of antimicrobial natural products. The detailed provenance information includes the producing organism, the culture conditions, and the isolation and characterization methods, providing a comprehensive view of each compound’s origin and discovery. These resources collectively provide the structural foundation for NP-GUARD’s candidate library.

On the antimicrobial side, CO-ADD (Community for Open Antimicrobial Drug Discovery) provides standardized screening data for compounds submitted by the research community [43]. The CO-ADD initiative was established to address the decline in antimicrobial drug discovery by providing free screening for academic researchers. The resulting dataset includes standardized activity measurements against a panel of bacterial and fungal pathogens, providing a consistent basis for model training and evaluation. The panel includes both Gram-positive and Gram-negative bacteria, covering the spectrum of clinically relevant pathogens. The standardization of the screening protocol ensures that the activity measurements are comparable across compounds, facilitating the development of predictive models.

ChEMBL is a comprehensive database of bioactivity data curated from the medicinal chemistry literature, providing quantitative measurements against thousands of targets and organisms [44]. The database includes information on compound structure, activity, target, and assay conditions, and is widely used in cheminformatics and drug discovery research. The database is updated regularly and includes data from both published studies and high-throughput screening campaigns. The quantitative activity data, including IC50, EC50, and MIC values, provide a rich source of labelled data for training and validating predictive models. The database also includes detailed information on assay conditions, enabling researchers to account for experimental heterogeneity in their analyses.

PubChem BioAssay provides a rich source of screening data from high-throughput screens and other bioactivity assays [45]. The database includes data from both public and private sources, providing a large and diverse collection of bioactivity measurements. The high-throughput screening data are particularly valuable for model training, as they include large numbers of compounds screened under standardized conditions. The database also includes detailed information on assay protocols and quality control measures, enabling researchers to assess the reliability of the activity measurements. Together, these resources provide the labelled antimicrobial activity data required for training and validation.

The heterogeneity of these resources poses substantial integration challenges: identical compounds may be represented using different identifiers, protonation states, salt forms, stereochemical conventions, and structure encodings, while antimicrobial activity may be reported using different experimental endpoints and units. For example, minimum inhibitory concentrations may be reported in μg/mL, μM, or as qualitative categories, requiring careful conversion to a common representation for model training. Similarly, compound structures may be represented as SMILES strings with different canonicalization conventions, requiring careful standardization to ensure that identical compounds are recognized as such. The challenge of structure standardization is particularly acute for natural products, which often contain complex stereochemistry and multiple chiral centers that can be represented in different ways. NP-GUARD addresses these challenges through a dedicated data curation layer (Layer 1) that performs explicit structure standardization, provenance tracking, and activity harmonization. Every imported observation is retained together with its source database, original identifier, source version, assay identifier, literature reference, and transformation history, ensuring that each label can be traced back to its primary measurement. This provenance-aware approach provides a reproducible foundation for model development and evaluation, enabling researchers to understand exactly what data were used and how they were processed.

### 2.7. Summary and Positioning of NP-GUARD

The preceding review highlights several critical gaps in current approaches to antimicrobial natural-product discovery. First, most models are trained and evaluated on a single pathogen and a single activity endpoint, ignoring the multidimensional nature of antimicrobial utility. A compound that is highly active against *S. aureus* but toxic to human cells, or one that is active in vitro but poorly soluble, is unlikely to become a viable therapeutic lead. Second, predictive uncertainty is rarely quantified or propagated into candidate ranking, leading to inefficient allocation of experimental resources. A molecule receiving an extreme but highly uncertain activity score is often treated equivalently to one with a robustly supported prediction, leading to wasted experimental effort on compounds that are unlikely to be active. Third, random train–test splitting overestimates generalization to novel scaffolds, and scaffold-aware evaluation is not routinely performed. The use of random splitting can inflate performance metrics by allowing near-identical analogues to appear in both training and test sets, providing an overly optimistic estimate of the model’s ability to discover novel chemotypes. Fourth, interpretability analyses are typically reported for a single fitted model without assessing stability across independently trained models. The instability of explanations can lead to misguided optimization efforts if the highlighted features are not reproducible. Fifth, natural products are underrepresented in training collections, leading to a critical domain-shift problem. The distinctive chemistry of natural products, including their stereochemical complexity and structural diversity, is not well captured by models trained primarily on synthetic compounds.

NP-GUARD is explicitly designed to address these gaps through a seven-stage architecture organized around six core computational modules, integrated within a provenance-aware knowledge base and user interface. The framework integrates provenance-tracked data curation, multi-view molecular representation, pathogen-conditioned multi-task prediction, explicit uncertainty quantification, stability-aware explainability, and conformal-Pareto prioritization, all consolidated within a queryable knowledge base. This design separates representation, prediction, trustworthiness, and decision-making, enabling the system to recommend compounds not solely on the basis of high predicted activity, but also on their reliability, novelty, and structural diversity. The framework represents a significant advance over conventional virtual-screening pipelines by reformulating antimicrobial natural-product discovery as an uncertainty-aware, relational, and multi-objective learning problem. The separation of concerns enables modular development and evaluation, allowing each component to be independently optimized and validated. The provenance-aware knowledge base provides a foundation for reproducible research, enabling other researchers to understand exactly what data were used and how they were processed. The framework is designed to be extensible, allowing new data sources, representation methods, and prediction models to be incorporated as they become available. NP-GUARD provides a transparent, reproducible, and decision-oriented foundation for prioritizing natural products for experimental validation, addressing the critical need for new antibiotics in the face of rising antimicrobial resistance.

## 3. Computational Architecture for Explainable and Uncertainty-Aware Natural-Product Prioritization

### 3.1. Overview and Design Principles

The computational component of the proposed framework is conceived not as a conventional virtual-screening pipeline based on a single classifier, but as a modular decision-support architecture for discovering structurally novel and experimentally actionable antimicrobial natural products. Recent studies, reviewed in Section 2, have demonstrated that deep molecular representations can identify antibacterial compounds that are structurally distant from established antibiotics [13,14,15], and that pretrained molecular representations transfer to antimicrobial prediction even when experimentally labelled datasets are small [19]. These developments motivate moving beyond descriptor-based Random Forest or gradient-boosting models as the principal methodological contribution. Although such methods remain important and will be retained as rigorous baselines, we propose a new architecture, NP-GUARD, designed around five core principles that collectively distinguish it from conventional virtual-screening approaches. First, the framework integrates heterogeneous natural-product and antimicrobial-assay resources with explicit provenance tracking, ensuring that every prediction can be traced back to its primary data source and that the entire workflow is fully reproducible. Second, it employs natural-product-aware molecular representation learning from complementary chemical views, i.e., graphs, SMILES strings, and interpretable physicochemical concepts, recognizing that no single representation captures all aspects of natural-product chemistry. Third, it implements pathogen-conditioned multi-task prediction rather than independent organism-specific classifiers, treating antimicrobial activity as a relationship between compounds and microorganisms. Fourth, it incorporates explicit modeling of predictive uncertainty, applicability domain, and explanation stability, ensuring that users know not only what the model predicts but also how reliable that prediction is. Fifth, it performs uncertainty-aware multi-objective prioritization that jointly accounts for antimicrobial efficacy, ADMET properties, toxicity, chemical novelty, and structural diversity, guiding experimental resources toward compounds that represent the most favorable trade-offs across these competing objectives.

The proposed architecture therefore separates *representation*, *prediction*, *trustworthiness*, and *decision making* into distinct, modular layers. This separation is important because the molecule receiving the highest predicted probability of activity is not necessarily the most appropriate experimental candidate. A molecule may receive a high activity score while lying far outside the chemical domain represented in the training set, exhibiting an unfavorable toxicity profile, or belonging to a large cluster of nearly identical analogues. NP-GUARD is explicitly designed to represent and propagate these different dimensions of evidence rather than collapsing them prematurely into a single score.

**Figure 1:**
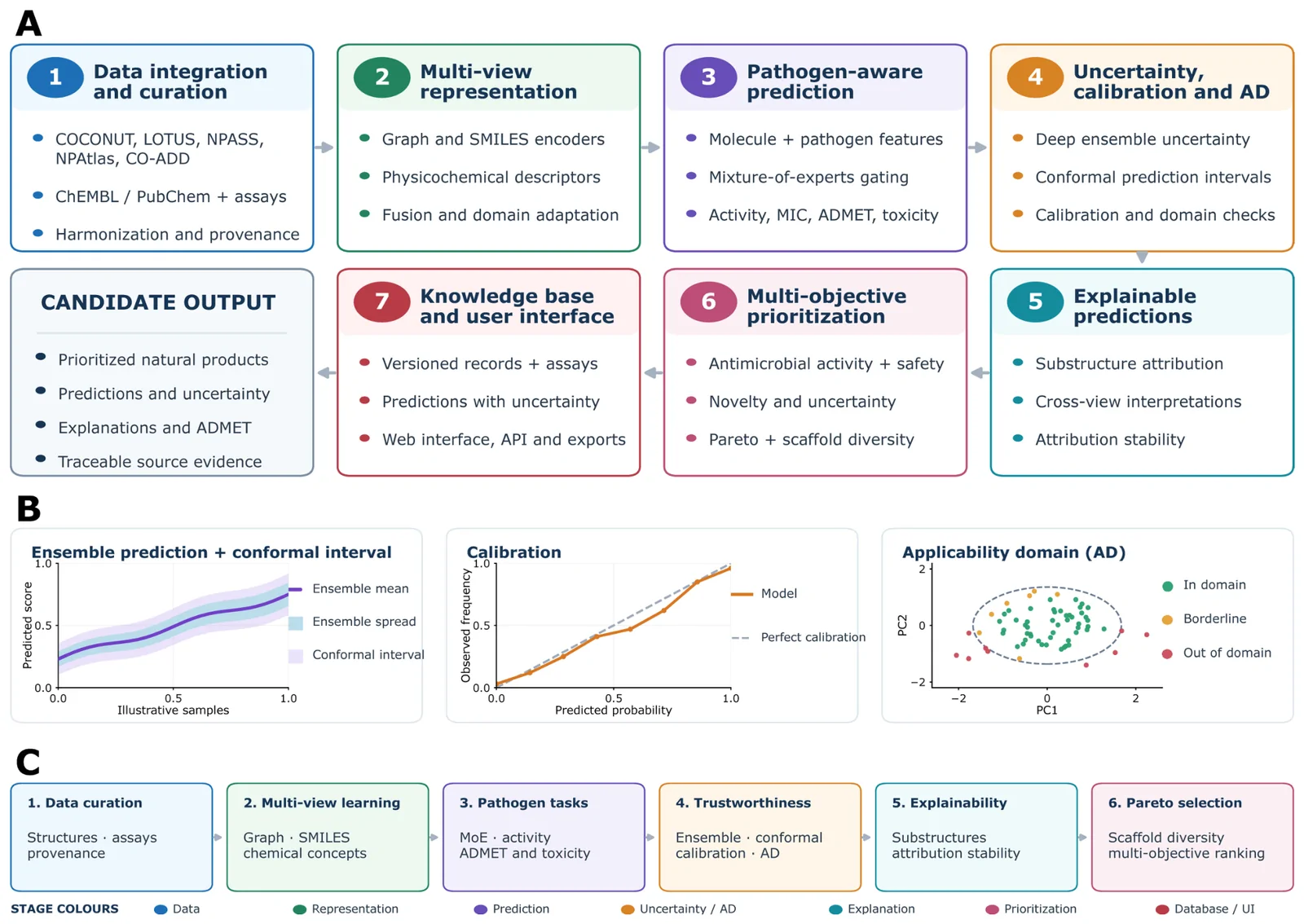
**Overview of the NP-GUARD framework architecture.** (**A**) Seven-stage architecture for pathogen-aware natural-product prioritization. The workflow integrates heterogeneous data from natural-product repositories, chemical databases, and biological assays through data curation, multi-view molecular representation learning, pathogen-aware prediction, uncertainty quantification and calibration, explainable predictions, multi-objective prioritization, and integration into a versioned knowledge base and user interface. The resulting candidate output includes prioritized natural products, activity predictions with uncertainty estimates, molecular explanations, ADMET information, and traceable source evidence. (**B**) Schematic illustrations of three model trustworthiness diagnostics. The ensemble prediction panel displays the ensemble mean as a solid purple line, with light-blue shading representing ensemble spread and light-purple shading representing the conformal prediction interval. The calibration panel compares model calibration (solid orange line) with perfect calibration (dashed gray line). The applicability domain panel distinguishes in-domain (green), borderline (orange), and out-of-domain (red) observations in an illustrative principal-component space. These plots are conceptual examples and do not represent measured model performance. (**C**) Six computational modules summarize the implementation pipeline: data curation, multi-view learning, pathogen-specific prediction tasks, trustworthiness assessment, explainability, and Pareto-based candidate selection. The color scheme distinguishes the functional stages: blue for data integration, green for molecular representation, purple for prediction, orange for uncertainty and applicability-domain assessment, teal for explainability, magenta for multi-objective prioritization, and red for the knowledge base and user interface. Together, these components support transparent, uncertainty-aware, and diversity-conscious prioritization of natural-product candidates.

Figure 1 illustrates the overall NP-GUARD architecture, organized into seven interconnected stages (Figure 1A) that progressively transform heterogeneous natural-product and antimicrobial-assay data into an interpretable, uncertainty-aware, and experimentally actionable candidate shortlist.

Stage 1 establishes the data foundation by integrating natural-product repositories, chemical databases, and antimicrobial screening resources through structure standardization, assay harmonization, and explicit provenance tracking. Stage 2 defines a natural-product- aware multi-view representation strategy that combines molecular graph encoders, SMILES-based chemical language models, and interpretable physicochemical descriptors through cross-modal fusion and domain adaptation. Stage 3 introduces pathogen-aware relational prediction, in which molecular and microorganism representations are jointly processed through a mixture-of-experts architecture to support antimicrobial activity prediction and related ADMET and toxicity tasks. Stage 4 addresses predictive trustworthiness through uncertainty quantification, probability calibration, conformal prediction, and applicability-domain assessment, allowing candidate predictions to be evaluated in relation to their statistical reliability and chemical-domain support. Stage 5 incorporates molecular explainability through substructure attribution, complementary representation-level interpretations, and formal assessment of explanation stability across independently fitted models. Stage 6 integrates these predictive and explanatory outputs into an uncertainty-aware multi-objective prioritization procedure, balancing antimicrobial activity, safety, chemical novelty, and scaffold diversity through conformal-Pareto selection principles. Finally, Stage 7 specifies the consolidation of compounds, assay information, model predictions, uncertainty estimates, and molecular explanations within a versioned, provenance-aware knowledge base accessible through an application programming interface (API) and web-based interface. The architecture is therefore designed to preserve traceability and uncertainty information throughout the transition from molecular evidence to candidate selection, rather than reducing the discovery process to a single activity prediction or composite ranking score.

Figure 1B provides a complementary conceptual representation of the trustworthiness mechanisms incorporated into the framework. The first diagnostic illustrates ensemble-based prediction and conformal uncertainty, distinguishing the ensemble mean from the variability across model predictions and the corresponding conformal prediction interval. The second depicts probability calibration by comparing model-predicted probabilities with observed event frequencies, thereby illustrating how departures from ideal calibration can be identified. The third represents applicability-domain assessment in an illustrative principal-component space, differentiating candidates located within the modeled chemical domain from borderline and out-of-domain observations. These complementary procedures address distinct aspects of predictive reliability: variability among fitted models, agreement between predicted probabilities and observed outcomes, and representational proximity to the chemical space supporting model development. Importantly, the diagnostic plots in Figure 1B are schematic illustrations of the proposed computational procedures and do not represent empirical performance measurements. Their inclusion emphasizes that uncertainty quantification, calibration, and applicability-domain assessment are complementary safeguards rather than interchangeable measures of prediction confidence. Their actual behavior must be established through independent calibration and evaluation, particularly under the scaffold-aware data partitions adopted in the present study.

Figure 1C summarizes the six principal computational modules underlying the seven-stage architecture: data curation and knowledge-base construction, multi-view molecular representation learning, pathogen-conditioned multi-task prediction, trustworthiness assessment, molecular explainability, and Pareto-based prioritization with chemical diversity selection. The distinction between seven architectural stages and six computational modules reflects their different levels of abstraction: the former describes the complete information-processing and delivery workflow, including the user-facing knowledge-base interface, whereas the latter identifies the principal computational functions required to transform molecular and assay evidence into prioritized candidates. The color coding and directional arrows indicate the functional organization and intended flow of information across the modules, while their modular separation permits individual components to be developed, evaluated, and refined without requiring simultaneous implementation of the complete architecture. This distinction is particularly relevant to the present study, which specifies the full NP-GUARD framework while reporting a proof-of-concept evaluation of selected data curation, predictive modeling, uncertainty, explainability, and prioritization components. The complete multi-view encoder, its natural-product domain-adaptation strategy, and the integrated knowledge-base interface remain components of the broader architectural specification requiring further implementation and validation. Consequently, the present evaluation should be understood as an assessment of selected computational mechanisms and their contribution to reproducible, uncertainty-aware compound prioritization, rather than as experimental validation of the entire end-to-end platform.

### 3.2. Layer 1: Heterogeneous Data Acquisition, Harmonization and Provenance

The first layer constructs a versioned natural-product knowledge base from complementary public resources, establishing the data foundation upon which all subsequent analyses depend. Candidate molecular structures can be obtained from large open natural-product collections including COCONUT [9], LOTUS [10], NPASS [11], and the Natural Products Atlas [12]. Antimicrobial activity records can additionally be integrated from resources such as CO-ADD [43], ChEMBL [44], PubChem BioAssay [45], and curated literature datasets. These resources contain partially overlapping but complementary information concerning chemical structure, biological origin, microbial activity, molecular targets, cytotoxicity, and pharmacokinetic properties.

Data integration represents a non-trivial computational component because identical compounds may be represented using different identifiers, protonation states, salt forms, stereochemical conventions, and structure encodings, while antimicrobial activity may be reported using different experimental endpoints and units. For example, minimum inhibitory concentrations may be reported in μg/mL, μM, or as qualitative categories, requiring careful conversion to a common representation for model training. Similarly, compound structures may be represented as SMILES strings with different canonicalization conventions, requiring careful standardization to ensure that identical compounds are recognized as such. The challenge of structure standardization is particularly acute for natural products, which often contain complex stereochemistry and multiple chiral centers that can be represented in different ways.

We therefore propose an explicit extract–transform–load workflow in which every imported observation is retained together with its source database, original identifier, source version, assay identifier, literature reference, and transformation history. This provenance-aware approach ensures that each label can be traced back to its primary measurement and that the entire data processing pipeline is fully transparent and reproducible.

Molecular structures will be processed using a reproducible standardization procedure. The raw and standardized representations will both be retained to preserve the original information while enabling consistent downstream analysis. The procedure will include molecular sanitization (ensuring that all atoms and bonds are chemically valid), explicit treatment of disconnected fragments and salts, canonical SMILES generation, InChI/InChIKey calculation, preservation of stereochemical information whenever available, and computation of Bemis–Murcko scaffolds [31]. Deduplication will be performed at several levels so that exact duplicates can be distinguished from stereoisomers, alternative protonation states, and closely related analogues. This multi-level deduplication is essential for ensuring that the training data are not contaminated by redundant records that could lead to overly optimistic performance estimates.

Antimicrobial measurements require comparable attention. Whenever possible, minimum inhibitory concentrations will be converted to a common molar representation and transformed into a logarithmic activity scale. Let ${\mathrm{MIC}}_{i}$ denote the minimum inhibitory concentration of compound *i*, expressed in molar units ($\mathrm{mol}\;L^{-1}$). The corresponding ${\mathrm{pMIC}}_{i}$ value is then defined as:

(1) $${\mathrm{pMIC}}_{i}=-\log_{10}\left(\frac{{\mathrm{MIC}}_{i}}{1\;\mathrm{mol}\;L^{-1}}\right).$$

This logarithmic transformation serves several purposes: it compresses the wide dynamic range of MIC values (which can span several orders of magnitude), provides a more symmetric distribution suitable for regression modeling, and aligns with standard practice in quantitative structure–activity relationship modeling. Importantly, measurements reported as inequalities, for example, MIC>64μg/mL or MIC<1μg/mL, will not be automatically converted to arbitrary point estimates. Instead, they will be retained as censored observations and handled using interval-aware loss functions. This approach preserves substantially more information than premature binarization, which would discard the quantitative information contained in the inequality thresholds.

Pathogen names will be mapped to normalized taxonomic identifiers and, where available, strain-level metadata. This is essential for enabling pathogen-conditioned prediction, as different strains of the same species may exhibit different susceptibility profiles. Assay information including microbial strain, growth medium, incubation conditions, assay type, and endpoint will be retained because apparently conflicting activities may reflect experimental heterogeneity rather than annotation error. For example, a compound that appears inactive against a particular species in one assay may be active under different conditions, and retaining this metadata enables the model to learn these assay-dependent effects.

Drug-likeness rules will primarily be represented as annotations and optimization objectives rather than hard filters. This design is particularly relevant for natural products, which frequently occupy chemical regions that differ from conventional synthetic medicinal-chemistry libraries. Natural products often violate rules such as Lipinski’s Rule of Five while still possessing valuable biological activity, and applying such rules as hard filters would systematically exclude many of the most interesting compounds. Except for invalid structures, duplicates, and clearly unusable records, potentially interesting compounds should not be removed before predictive analysis solely because they violate conventional heuristic rules.

### 3.3. Layer 2: Natural-Product-Aware Multi-View Molecular Representation

The central representation layer moves beyond the assumption that a single molecular fingerprint or descriptor vector provides a sufficient representation of natural-product chemistry. A molecule $x_{i}$ is instead represented through complementary views:

$$x_{i}=\left(G_{i},S_{i},C_{i}\right),$$

where $G_{i}$ denotes its molecular graph, $S_{i}$ its string-based SMILES representation, and $C_{i}$ a vector of interpretable physicochemical and structural concepts. This multi-view approach recognizes that different representations capture different aspects of molecular structure and that combining them can provide a more complete description of the chemical features relevant to antimicrobial activity.

At the core of the representation layer lies the graph-based view, which captures the molecular structure in a form that directly mirrors chemical intuition. The molecular graph is defined as $G_{i}=\left(V_{i},E_{i}\right)$, where atoms correspond to nodes $V_{i}$ and covalent bonds to edges $E_{i}$. Atom-level attributes include atomic number, formal charge, aromaticity, hybridization, chirality, and ring membership, while edge attributes include bond type, conjugation, aromaticity, and stereochemistry. This rich set of features enables the model to capture the detailed structural information required for distinguishing between subtly different chemical compounds. Rather than relying exclusively on conventional graph convolution, we propose an edge-aware Graph Transformer combining local message passing with global self-attention. Message passing captures the local chemical environment around atoms and functional groups, propagating information through the molecular graph in a way that mirrors the propagation of electronic effects in molecules. Transformer-style attention provides a mechanism for representing longer-range relationships between molecular substructures, enabling the model to capture interactions between distant parts of the molecule that may be important for biological activity. Large-scale self-supervised graph models such as GROVER have demonstrated the potential of graph-transformer pretraining for molecular representation learning [36], while recent hybrid GCN–Transformer architectures further support the integration of local and global molecular information. The graph encoder produces the representation $z_{i}^{G}=E_{G}\left(G_{i}\right)\in R^{d_{G}}$, where $E_{G}$ denotes the graph encoder network and $d_{G}$ is the dimensionality of the resulting embedding, which captures the structural features of the molecule as encoded in its graph topology and atomic attributes.

A second branch represents the same molecule using a pretrained chemical language model operating on its SMILES representation. Transformer models such as ChemBERTa and MolFormer have demonstrated that large-scale self-supervised learning from unlabeled chemical sequences can generate transferable molecular representations [17,35]. MolFormer, for example, was trained at very large scale and demonstrated that SMILES language models can capture chemical and structural information useful across heterogeneous downstream molecular-property tasks. The underlying principle is that pretraining on a large corpus of chemical structures allows the model to learn general features of molecular chemistry that can be fine-tuned for specific prediction tasks. The SMILES branch produces $z_{i}^{S}=E_{S}\left(S_{i}\right)\in R^{d_{S}}$, where $E_{S}$ denotes the SMILES encoder and $d_{S}$ is the dimensionality of the SMILES embedding. Multiple randomized SMILES strings corresponding to the same molecular structure can be used during training as data augmentation, thereby discouraging the model from learning artifacts associated with one particular string serialization. This augmentation strategy leverages the fact that the same molecule can be represented by multiple valid SMILES strings due to the flexibility of the SMILES grammar, and training on multiple augmentations encourages the model to learn representations that are invariant to the specific serialization.

The third branch contains descriptors whose chemical meaning can be explicitly communicated to users. These may include molecular weight, topological polar surface area, hydrogen-bond donors and acceptors, calculated lipophilicity, formal charge, fraction of sp^3^ carbons, aromaticity, ring statistics, rotatable bonds, and selected natural-product-relevant structural descriptors. These features are derived from fundamental chemical principles and provide interpretable, chemically meaningful summaries of molecular properties. This representation is denoted as $z_{i}^{C}=E_{C}\left(C_{i}\right)$, where $E_{C}$ is a feature encoder and $C_{i}$ is the vector of interpretable physicochemical descriptors for compound *i*. This explicit concept branch has two purposes: first, conventional chemical features can contribute predictive information complementary to learned embeddings, particularly when the available data are limited, as many of these features have established relationships with biological activity; second, the concept branch provides a natural interface between deep representation learning and global model explainability, enabling users to understand which physicochemical properties drive predictions.

As an optional extension, natural products frequently contain complex stereochemistry and conformationally constrained structures that may not be fully represented by two-dimensional topology. A three-dimensional molecular representation obtained from generated conformers and a pretrained geometric model such as Uni-Mol [18] can be incorporated. The integration of 3D information is particularly relevant for natural products, which often exhibit complex stereochemistry that influences their biological activity through specific interactions with chiral biological targets. Because conformer generation and 3D inference introduce additional computational cost and uncertainty, the 3D branch should initially be evaluated as an ablation rather than assumed a priori to improve prediction.

### 3.4. Natural-Product-Specific Domain Adaptation

A distinguishing component of NP-GUARD is that pretrained representations are not used only in their generic form. General chemical foundation models are trained predominantly on very large heterogeneous chemical spaces, whereas natural products show characteristic distributions of stereochemical complexity, ring systems, oxygenation patterns, and scaffold architectures. This domain shift means that representations learned on the general chemical corpus may not be optimal for natural-product applications. We therefore propose a second self-supervised adaptation stage performed on the unlabeled natural-product corpus assembled from COCONUT, LOTUS, NPASS, and related resources. The purpose is not to learn antimicrobial labels but to adapt the representation space to natural-product chemistry before supervised antimicrobial training, analogous to domain adaptation in computer vision.

For the graph and language branches, the domain-adaptation objective is formulated as:

$$L_{NP}=\lambda_{G}L_{graph}+\lambda_{S}L_{SMILES}+\lambda_{A}L_{align},$$

where $L_{graph}$ represents masked atom/bond or graph-context reconstruction, encouraging the graph encoder to learn representations that capture the structural patterns of natural products; $L_{SMILES}$ represents masked-token or augmented-SMILES learning, performing a similar function for the language branch; and $L_{align}$ encourages graph and SMILES representations derived from the same molecule to occupy compatible regions of latent space, ensuring that the two views provide consistent information. The hyperparameters $\lambda_{G}$, $\lambda_{S}$, and $\lambda_{A}$ control the relative contribution of each objective. For a minibatch of molecules, cross-view alignment can be implemented through a contrastive objective in which the graph and SMILES encodings of the same compound constitute positive pairs and encodings from different compounds constitute negatives. This provides a principled mechanism for transferring information between two chemically equivalent but representationally distinct descriptions, yielding a natural-product-adapted cross-modal molecular representation specifically tuned to the distinctive chemistry of natural products.

### 3.5. Cross-Modal Representation Fusion

The graph, language, and concept embeddings are combined by a learnable gated fusion layer rather than simple vector concatenation. Given representations $z_{i}^{G}$, $z_{i}^{S}$, and $z_{i}^{C}$, modality-specific weights are calculated as:

$$\alpha_{i}=\mathrm{softmax}\left[g\left(z_{i}^{G},z_{i}^{S},z_{i}^{C}\right)\right],$$

where *g* is a small neural network that takes the concatenated representations as input and outputs a vector of three unnormalized scores, which are then transformed into a probability distribution over modalities via the softmax function. The integrated compound representation becomes:

$$z_{i}=\sum_{m\in \{G,S,C\}}\alpha_{im}W_{m}z_{i}^{m},$$

where $W_{m}$ are learnable projection matrices that transform each modality-specific embedding into a common representation space, and $\alpha_{im}$ are the modality weights. This formulation allows the model to learn that different molecular views may provide different amounts of information for different compounds. For example, for a compound with a well-defined graph structure but a SMILES representation that is difficult to parse, the model may learn to weight the graph representation more heavily, while for a compound whose graph structure is ambiguous but whose SMILES representation is clear, the SMILES branch may receive higher weight. Importantly, modality weights can subsequently be inspected as an additional measure of model behavior, providing insights into how different representations contribute to predictions. Simple descriptor/fingerprint concatenation, learned embeddings without fusion, and individual graph or language encoders will all be retained as ablation models.

### 3.6. Layer 3: Pathogen-Conditioned Relational Prediction

A second major departure from conventional antimicrobial QSAR modeling concerns the formulation of the prediction problem itself. Rather than fitting independent functions $f_{SA}\left(x\right)$, $f_{PA}\left(x\right)$, and $f_{EC}\left(x\right)$ for *S. aureus*, *P. aeruginosa*, and *E. coli*, we propose learning the relation f(x,p), where *x* represents a compound and *p* represents a pathogen. This formulation treats antimicrobial activity as a compound–pathogen interaction problem, allowing the model to learn molecular determinants shared across bacterial species while simultaneously representing organism-specific activity, analogous to matrix completion approaches in collaborative filtering.

Each target microorganism is associated with a pathogen representation $e_{p}=E_{P}\left(p\right)$, where $E_{P}$ is a pathogen encoder. In the initial implementation, $e_{p}$ can combine a learned species embedding with interpretable microbial attributes including Gram status and taxonomic information. If sufficiently broad antimicrobial data are obtained, the pathogen encoder can subsequently incorporate genomic or protein-derived representations, enabling the architecture to exploit similarity between related microorganisms. The molecular and pathogen representations are passed to a pathogen-conditioned Mixture-of-Experts layer. If *K* molecular experts are denoted by $E_{k}$, the pathogen-dependent gate is:

$$\alpha_{pk}=\frac{\exp(w_{k}^{⊤}e_{p})}{\sum_{j=1}^{K}\exp\left(w_{j}^{⊤}e_{p}\right)},$$

where $w_{k}$ are learnable weight vectors that determine the relevance of each expert to a given pathogen. The pathogen-specific molecular representation becomes:

$$h_{ip}=\sum_{k=1}^{K}\alpha_{pk}E_{k}\left(z_{i}\right),$$

and the final antimicrobial prediction is:

$${\hat{y}}_{ip}=H_{A}\left(h_{ip},e_{p}\right),$$

where $H_{A}$ is a prediction head. The interpretation is straightforward: the molecular encoder is shared, while the pathogen embedding determines which latent experts are most relevant for the particular organism. This provides a compromise between a single universal antimicrobial classifier and completely independent organism-specific models. Where quantitative MIC data are available, antimicrobial prediction will preferably be formulated jointly as regression and classification, using a multi-task loss that combines both objectives.

### 3.7. Masked Multi-Task Learning and Auxiliary Pharmacological Endpoints

Natural-product databases are intrinsically incomplete: one molecule may have an MIC measurement against *S. aureus* but no measurement against *P. aeruginosa*; another may contain cytotoxicity but no pharmacokinetic measurements. Requiring complete labels would discard a substantial fraction of the available data. Furthermore, the mechanisms that make a compound antimicrobial often also determine its toxicity and pharmacokinetic properties, and learning these relationships jointly can improve prediction accuracy for all tasks. NP-GUARD therefore adopts masked multi-task learning. Let $\Omega_{t}$ denote the compounds possessing an experimentally observed label for task *t*. The supervised objective is:

$$L_{sup}=\sum_{t=1}^{T}\lambda_{t}\frac{1}{|\Omega_{t}|}\sum_{i\in \Omega_{t}}\ell_{t}\left(y_{it},{\hat{y}}_{it}\right),$$

where *T* is the total number of tasks, $\lambda_{t}$ is a task-specific weighting factor, $|\Omega_{t}|$ denotes the number of compounds with labels for task *t*, $\ell_{t}$ is the loss function for task *t*, and $y_{it}$ and ${\hat{y}}_{it}$ are the true and predicted labels. Only experimentally available labels contribute to each task loss. This masking strategy enables the model to learn from all available data without requiring complete labels for all compounds, analogous to masking strategies used in transformer-based language models. Tasks may include pathogen-specific antimicrobial activity, quantitative MIC, cytotoxicity, hemolysis, solubility, permeability, metabolic stability, and other ADMET endpoints. NPASS 3.0 is particularly relevant because its current release substantially extends experimentally annotated activity, ADME, and toxicity information [11]. Task-specific adapters or prediction heads are used above the common molecular representation so that shared chemical information can be exploited without forcing all endpoints to use an identical decision function.

### 3.8. Layer 4: Predictive Uncertainty, Calibration and Applicability Domain

A candidate-ranking system intended to guide experimental resources must distinguish a strong prediction from a confident prediction. Deep neural models can assign high scores even to molecules lying far outside the distribution represented by the training data. Accordingly, uncertainty estimation is treated as a first-class model output, integrated into the prioritization process rather than added as an afterthought. We propose combining deep ensembles with conformal prediction.

Multiple independently initialized models are trained within each training partition, providing an empirical measure of epistemic variability. For regression endpoints, the ensemble mean and variance provide point estimates and uncertainty intervals:

$${\hat{\mu }}_{i}=\frac{1}{M}\sum_{m=1}^{M}{\hat{y}}_{i}^{\left(m\right)}\;\mathrm{and}\;\sigma_{i}^{2}=\frac{1}{M-1}\sum_{m=1}^{M}{\left({\hat{y}}_{i}^{\left(m\right)}-{\hat{\mu }}_{i}\right)}^{2},$$

where *M* is the number of ensemble members, ${\hat{y}}_{i}^{\left(m\right)}$ is the prediction of the *m*-th ensemble member, ${\hat{\mu }}_{i}$ is the ensemble mean, and $\sigma_{i}^{2}$ is the ensemble variance. Deep ensembles have been shown to outperform more complex Bayesian methods in many practical settings while being computationally simpler to implement [28]. However, ensemble variance alone does not provide a finite-sample coverage guarantee. Conformal prediction will therefore be applied using calibration observations drawn exclusively from the training data [26]. For MIC regression, the system consequently reports an interval $\left[L_{i},U_{i}\right]$ rather than only ${\hat{y}}_{i}$, while for classification, calibrated probabilities and conformal prediction sets can be generated. Probability calibration, including temperature scaling, Platt scaling, or isotonic regression, will always be fitted within the training portion of the corresponding resampling iteration. No test-set outcome will be used for calibration or threshold selection.

Applicability domain is evaluated independently of predictive uncertainty. A simple structure-based component is:

$$AD_{i}^{struct}=\max_{j\in D_{train}}\mathrm{Tanimoto}\left(FP_{i},FP_{j}\right),$$

where FP denotes an ECFP representation, quantifying the maximum similarity between the candidate compound and any training compound. This can be complemented by a distance in learned representation space:

$$AD_{i}^{emb}=\exp\left(-\frac{d(z_{i},D_{train})}{\tau }\right),$$

where $d(z_{i},D_{train})$ is the distance from the candidate’s learned representation to the training set distribution and τ is a temperature parameter. The combined applicability score permits explicit identification of molecules that are structurally or representationally distant from the training set. Importantly, such compounds should not automatically be discarded, a high-potential out-of-domain natural product may represent precisely the structural novelty being sought, but should instead be labelled as requiring greater experimental caution.

### 3.9. Layer 5: Multi-Resolution Explainable Artificial Intelligence

Explainability is integrated at multiple representational levels rather than treated as a single post-hoc visualization, enabling different types of explanations for different audiences and purposes. At the molecular-concept level, SHAP (SHapley Additive exPlanations) values quantify the contribution of interpretable physicochemical variables to antimicrobial and ADMET predictions [38], providing global information about which properties, such as lipophilicity, charge, aromaticity, or polar surface area, systematically influence predictions. These explanations are accessible to chemists and medicinal chemists, as they directly relate to chemical properties that can be modified during compound optimization. At the molecular-graph level, Integrated Gradients [40], GNNExplainer [41], or related graph-attribution strategies can assign contributions to atoms, bonds, and connected molecular subgraphs. Integrated Gradients computes attributions by integrating the gradients of the prediction with respect to the input along a path from a baseline to the input, satisfying several desirable axioms including sensitivity and implementation invariance. GNNExplainer optimizes a mask over edges and node features to find the smallest subgraph that is most influential for the prediction. Together, these methods permit the model to produce chemically interpretable rationales rather than only feature rankings, highlighting specific ring systems or functional groups responsible for predicted antimicrobial activity.

This distinction is important. For a prioritized molecule, the system should ideally answer both which physicochemical characteristics contribute to the prediction and which atoms or molecular substructures are primarily responsible for the predicted antimicrobial activity or toxicity. The second question is particularly relevant because explainable graph models have already demonstrated that model-derived molecular rationales can support discovery of structurally distinct antibiotic classes [15]. We further propose evaluating explanation stability, as an attribution obtained from a single trained network can be unstable even when the prediction itself is stable. Explanations will therefore be calculated across ensemble members and resampling folds. For the top-*k* explanatory features or substructures identified in runs *r* and *s*, stability is summarized as:

$$Stability_{k}=\frac{2}{R(R-1)}\sum_{r<s}J\left(S_{r}^{\left(k\right)},S_{s}^{\left(k\right)}\right),$$

where *R* is the number of independent runs, $S_{r}^{\left(k\right)}$ is the set of the top-*k* features identified in run *r*, and *J* denotes the Jaccard similarity coefficient. This metric quantifies the consistency of top-*k* explanations across independently trained models, with values ranging from 0 (no overlap) to 1 (identical sets). Only explanations demonstrating sufficient recurrence will be treated as robust mechanistic hypotheses, converting XAI from a purely illustrative component into an experimentally assessable property of the computational framework.

### 3.10. Layer 6: Conformal-Pareto Multi-Objective Prioritization

The output of the predictive layer comprises multiple quantities rather than a single antimicrobial score. For compound $x_{i}$, let:

$$f\left(x_{i}\right)=\left[A_{SA},A_{PA},A_{EC},D,-T,N,-U\right],$$

where $A_{SA}$, $A_{PA}$, and $A_{EC}$ represent predicted antimicrobial activity against the three target pathogens, *D* represents desirable ADMET behavior, *T* represents toxicity, *N* represents structural novelty, and *U* represents predictive uncertainty. Conventional weighted ranking would define $Score_{i}=\sum_{j}w_{j}f_{j}\left(x_{i}\right)$, but this immediately imposes arbitrary exchange rates between objectives. We therefore propose Pareto optimization as the primary selection principle [42]. A molecule is Pareto-optimal if no alternative candidate is at least as favorable for every objective and strictly superior for at least one. The Pareto front provides a principled way to identify compounds that represent optimal trade-offs without requiring arbitrary weights.

An important methodological extension is to perform this comparison using uncertainty-adjusted objectives. For beneficial endpoint *j*, let $L_{ij}$ be the lower conformal confidence bound, whereas for undesirable endpoint *k*, such as toxicity, let $U_{ik}$ be the upper confidence bound. The robust objective vector becomes:

$$f^{rob}\left(x_{i}\right)=\left[L\left(A_{SA}\right),L\left(A_{PA}\right),L\left(A_{EC}\right),L\left(D\right),-U\left(T\right),N,AD\right],$$

where AD denotes the applicability domain score. Thus, a compound does not become Pareto-optimal merely because it has an extreme but highly uncertain point prediction. High antimicrobial activity contributes strongly to selection only when supported by a sufficiently favorable lower confidence bound, while toxicity is evaluated conservatively using its upper uncertainty bound. This strategy, termed conformal-Pareto prioritization, directly links predictive uncertainty to the downstream experimental decision. For user-facing exploration, compounds within or close to the robust Pareto front can subsequently receive an optional composite score, with sensitivity analyses varying the associated weights to determine whether candidate ordering is robust to plausible changes in decision preferences.

### 3.11. Structural Novelty and Diversity-Aware Candidate Selection

An antimicrobial-screening algorithm that returns twenty close analogues of the same scaffold provides considerably less discovery value than one returning a chemically diverse set of high-quality candidates. Structural novelty relative to the labelled training set is defined as:

$$N_{i}=1-\max_{j\in D_{train}}\mathrm{Tanimoto}\left(FP_{i},FP_{j}\right),$$

where $FP_{i}$ and $FP_{j}$ are ECFP fingerprints of the candidate and a training compound, respectively, and Tanimoto denotes the Tanimoto similarity coefficient. This metric ranges from 0 (identical to a training compound) to 1 (maximally dissimilar). Bemis–Murcko scaffold identity provides a complementary discrete measure of scaffold novelty. Candidate compounds will additionally be clustered in fingerprint or learned-representation space. Selection from the Pareto frontier then uses a diversity-aware top-*k* algorithm. After selecting candidate set S, the next compound is chosen according to:

$$x^{*}=\arg\max_{x\notin S}\left[\rho R\left(x\right)-\left(1-\rho \right)\max_{s\in S}\mathrm{Sim}\left(x,s\right)\right],$$

where R(x) is the uncertainty-adjusted prioritization score, Sim(x,s) represents chemical similarity, and ρ∈[0,1] controls the trade-off between quality and diversity. When ρ=1, the algorithm selects purely based on quality; when ρ=0, it selects based purely on diversity. This procedure favors compounds that are individually strong while penalizing redundancy with previously selected candidates, ensuring the final shortlist samples different regions of high-priority chemical space.

### 3.12. Layer 7: Provenance-Aware Computational Knowledge Base

The outputs of the workflow will be represented in a versioned computational knowledge base rather than a static results table. The core entities include Compound, Natural-Product Source, Organism, Antimicrobial Assay, Pathogen, ADMET Measurement, Model Prediction, Uncertainty Estimate, Molecular Explanation, Structural Cluster, and Prioritization Result. A relational implementation based on PostgreSQL with chemical extensions can support exact structure, substructure, and molecular-similarity queries, while vector indexing can support nearest-neighbor retrieval directly in the learned NP-GUARD embedding space.

For each prediction, the knowledge base will store a structured record comprising the following tuple:

$$R_{i}=\left(\mathrm{compound}\_\mathrm{id},\mathrm{endpoint},\mathrm{model}\_\mathrm{version},\mathrm{data}\_\mathrm{version},{\hat{y}}_{i},{\hat{p}}_{i},u_{i},{\mathrm{AD}}_{i},E_{i}\right),$$

where compound_id is the unique identifier of the compound; endpoint specifies the predicted property; model_version and data_version record the exact model and data versions used; ${\hat{y}}_{i}\in R$ is the raw model prediction; ${\hat{p}}_{i}\in \left[0,1\right]$ is the post-calibration probability estimate; $u_{i}\in R_{\geq 0}$ quantifies the predictive uncertainty; ${\mathrm{AD}}_{i}\in \left[0,1\right]$ is the applicability domain score; and $E_{i}$ stores the molecular explanation. This comprehensive record enables full reproducibility, as each prediction can be traced back to the specific model and data version that generated it. The database interface should permit queries such as: “identify natural products predicted to inhibit *P. aeruginosa*, with low predicted mammalian toxicity, a novel scaffold, a lower confidence bound above a specified activity threshold, and Tanimoto similarity below 0.4 to known training compounds.” An API layer can expose the same operations programmatically, while the web interface can provide structure-, similarity-, source-organism-, pathogen-, property-, and explanation-based exploration. All rankings remain linked to the exact model and database snapshot used to generate them, ensuring reproducibility and traceability.

### 3.13. Validation Framework and Diversity-Aware Prioritization

Rigorous validation is essential because random molecular splitting can substantially overestimate the ability of a model to generalize to novel chemical structures. Scaffold splitting provides a more challenging and discovery-relevant evaluation by separating compounds according to their core molecular frameworks [29]. The primary evaluation of NP-GUARD will therefore use repeated scaffold-aware splitting. All preprocessing operations that learn from data, including feature normalization, feature selection, hyperparameter optimization, probability calibration, conformal calibration, and decision-threshold determination, will be fitted exclusively within training data. Where database size permits, we further propose a scaffold-and-source external validation, holding out compounds originating from one data source or literature period to determine whether the model generalizes not merely to unseen molecular scaffolds but also to measurements collected outside the dominant training resource. Random splitting may still be reported because it facilitates comparison with previous literature, but it will be treated as a secondary benchmark rather than the principal estimate of discovery performance.

The proposed architecture must demonstrate that its additional complexity provides measurable value. NP-GUARD will therefore be evaluated against progressively stronger baselines. The first level consists of conventional descriptor and fingerprint models, including Random Forest, Gradient Boosting, and XGBoost. The second level consists of established molecular deep-learning methods such as D-MPNN and a standalone graph neural network. A pretrained-representation baseline inspired by MolE will determine whether generic self-supervised molecular representations combined with a conventional classifier can already explain the observed performance [19]. Standalone Graph Transformer and chemical-language-model variants will then be compared with the proposed cross-modal representation.

The principal ablations will assess the contribution of each component in a stepwise manner:

$$\begin{array}{cc} \mathrm{Graph}\;\mathrm{only} & \to \mathrm{Graph}\;+\;\mathrm{SMILES}\to \mathrm{Graph}\;+\;\mathrm{SMILES}\;+\;\mathrm{concepts}\\ \; & \to \mathrm{NP}\;\mathrm{domain}\;\mathrm{adaptation}\to \mathrm{pathogen}\;\mathrm{conditioning}\to \mathrm{multi}\text{-}\mathrm{task}\;\mathrm{learning}. \end{array}$$

Each arrow indicates the addition of a new component, starting from a baseline using only graph representations and successively adding the SMILES branch, the interpretable concept branch, natural-product-specific domain adaptation, pathogen-conditioned prediction, and finally multi-task learning. Separate experiments will evaluate uncertainty quantification, explanation stability, and Pareto/diversity-aware ranking.

For antimicrobial classification, performance will be summarized using AUROC, AUPRC, Matthews correlation coefficient, balanced accuracy, sensitivity, and specificity, with particular attention to AUPRC and MCC given the strong class imbalance typical of antimicrobial datasets. Calibration will be evaluated using the Brier score, expected calibration error, and reliability curves. Quantitative MIC models will be evaluated using MAE, RMSE, and rank correlation. For the final prioritization problem, we will quantify enrichment of known active compounds among the top-ranked candidates, scaffold diversity, mean pairwise chemical similarity, proportion of previously unseen scaffolds, and stability of the candidate ranking across resampling runs. Pareto hypervolume can provide a further summary of the quality and diversity of multi-objective candidate sets.

### 3.14. Proposed Computational Novelty

The principal computational contribution of the proposed study is therefore not the application of an individual machine-learning classifier to natural-product descriptors, but rather the reformulation of antimicrobial natural-product discovery as an uncertainty-aware relational and multi-objective learning problem. NP-GUARD combines a natural-product-adapted multi-view molecular encoder with pathogen-conditioned prediction, allowing antimicrobial activity to be learned as a relationship between compounds and microorganisms. The architecture jointly models antimicrobial activity and pharmacological properties while retaining incomplete experimental labels through masked multi-task training. Prediction reliability is explicitly estimated using ensemble and conformal methods, and interpretability is assessed not only through individual explanations but also through their stability across independently fitted models. Finally, the candidate-ranking stage introduces uncertainty directly into multi-objective decision making through conformal-Pareto prioritization, followed by diversity-aware selection. Consequently, a candidate is favored not simply because its expected antimicrobial activity is high, but because it represents a favorable, sufficiently reliable, and structurally informative trade-off between efficacy, safety, pharmacological suitability, and chemical novelty. This architecture is intended to transform conventional in silico screening from a ranking of molecular prediction scores into a transparent and reproducible framework for deciding which natural products should be experimentally tested next, why they should be tested, and how confident the computational evidence supporting that decision actually is.

## 4. Materials and Methods: Proof-of-Concept Evaluation

The architecture described in Section 3 defines the full NP-GUARD design, encompassing seven integrated layers spanning data acquisition, molecular representation learning, pathogen-conditioned prediction, uncertainty quantification, explainability, multi-objective prioritization, and provenance-aware knowledge management. Here we describe a proof-of-concept instantiation that implements and evaluates a subset of these components, specifically, data curation (Layer 1), predictive modelling (a per-endpoint instantiation of Layer 3 without pathogen conditioning), uncertainty quantification (Layer 4), explainability (Layer 5), and prioritization (Layer 6), using public data and deliberately simple baseline models, so that the evaluation protocol itself, rather than model complexity, is the primary object of study. The full multi-view molecular encoder with domain adaptation (Layer 2), pathogen-conditioned multi-task prediction (Layer 3), and provenance-aware knowledge base (Layer 7) are specified in Section 3 and will be evaluated in subsequent work.

No new laboratory experiments, physical instruments, or chemical reagents were used in this computational analysis; the datasets were obtained from previously published screening studies. Software versions and computational reproducibility details are provided in the Data Availability Statement.

### 4.1. Data Sources, Standardization, and Provenance

Four experimentally measured endpoints were assembled from public resources (Table 1), selected to represent complementary data regimes: prospective high-throughput screens (*S. aureus* and *E. coli*), cytotoxicity assessment across three human cell lines, and a literature-curated dataset (*P. aeruginosa*) that reflects the heterogeneity and publication bias of aggregated bioactivity databases.

The primary screen for *S. aureus* growth inhibition and human cytotoxicity was derived from Wong and colleagues [15], in which 39,312 compounds were tested at 10 µM against methicillin-susceptible *S. aureus* and, in parallel, against three human cell lines (HepG2 hepatocytes, primary human skeletal muscle cells, and IMR-90 lung fibroblasts). A compound was labelled active when its mean growth inhibition at 10 µM was ≥0.8, matching the hit threshold of the original study, and cytotoxic when the maximum mean inhibition across the three human cell lines was ≥0.5. This dual labelling strategy enables multi-objective prioritization that balances antimicrobial efficacy against safety.

The *E. coli* growth inhibition data were obtained from the primary screen of 2335 compounds reported by Stokes and colleagues [13], using the curated redistribution of the same data; the binary activity label of the original study was retained. Despite its modest size, this dataset is historically significant as it formed the basis for the discovery of halicin, a broad-spectrum antibiotic with a novel mechanism of action [13], and provides a stringent test of the evaluation protocol’s ability to assess generalization on limited data.

For *P. aeruginosa*, minimum inhibitory concentration (MIC) measurements were extracted from ChEMBL [44] (organism target CHEMBL348), yielding 58,498 activity rows. Measurements were harmonized to molar units and expressed as $p\mathrm{MIC}=-\log_{10}\left(\mathrm{MIC}\right)$, where MIC is expressed in molar units. This logarithmic transformation compresses the wide dynamic range of MIC values and aligns with standard practice in quantitative structure–activity relationship modeling. A compound was labelled active when its median pMIC corresponded to MIC ≤16 µg/mL. Censored measurements were handled explicitly: right-censored records (“MIC >x”) were labelled inactive only when x≥ the cutoff; left-censored records (“MIC <x”) were labelled active only when x≤ the cutoff; and records whose censoring interval contained the cutoff (870 compounds) were discarded as ambiguous. Because ChEMBL aggregates published medicinal-chemistry campaigns, the resulting active fraction (33.4%) reflects literature publication bias rather than an unbiased screening hit rate; we therefore treat this endpoint as a literature-mining benchmark.

The natural-product screening library was constructed from the COCONUT release of September 2026 [9] (738,827 structures). After standardization and de-duplication we retained all compounds with a deposited common name plus a reproducible random sample of 100,000 additional structures (seed 42), yielding a screening library of 406,959 unique natural products. Compounds whose deposited names match a curated list of known antibiotics (588 compounds) were flagged and used exclusively for enrichment evaluation, never for training.

All structures were processed with a single RDKit-based pipeline [46]: sanitization, retention of the largest fragment, reionization, neutralization of formal charges, canonical SMILES generation, and de-duplication by standard InChIKey. Bemis–Murcko scaffolds [31] were computed for every compound and used both for scaffold-aware splitting and for diversity analysis. Every curated record retains its source database, source identifier, raw activity value, units, censoring status, and standardization status, ensuring that each label can be traced back to its primary measurement (Layer 1 provenance). This provenance-aware approach guarantees full transparency and reproducibility.

### 4.2. Molecular Features and Baseline Models

Two complementary feature views were concatenated into a 2059-dimensional vector: (i) Morgan circular fingerprints (radius 2, 2048 bits), and (ii) eleven physicochemical descriptors (molecular weight, cLogP, topological polar surface area, hydrogen-bond donors and acceptors, rotatable bonds, fraction of sp^3^ carbons, aromatic ring count, ring count, heavy atom count, and QED drug-likeness). The Morgan fingerprints capture molecular topology, while the physicochemical descriptors capture global properties that influence drug-likeness and bioavailability.

Two gradient-boosting/forest baselines were trained per endpoint: Random Forest [47] (500 trees, balanced sub-sampling of classes, minimum two samples per leaf) and XGBoost [48] (800 trees, depth 6, learning rate 0.05, row sub-sampling 0.8, column sub-sampling 0.5, positive-class weighting by the training prevalence ratio), both implemented in scikit-learn [49]. As a deep-learning baseline, a directed message-passing neural network (D-MPNN) was trained with Chemprop 2.3.1 [16,34] using default architecture settings (300-dimensional bond messages, depth 3), 30 epochs, batch size 64, and scaffold-balanced splitting. The D-MPNN was included to assess whether deep learning provides additional predictive power beyond the fingerprint-based gradient-boosting models.

To implement the masked multi-task prediction layer specified in Section 3.7, a single D-MPNN with four classification heads (*S. aureus*, *E. coli* and *P. aeruginosa* activity, and cytotoxicity) was trained on the union of the four curated endpoint tables (68,818 unique compounds by InChIKey; labelled rows per endpoint: 38,995 *S. aureus*, 2291 *E. coli*, 29,986 *P. aeruginosa*, 38,996 cytotoxicity), with unobserved labels masked out of the training loss. Architecture, training epochs, batch size, scaffold-balanced 80/10/10 splitting and seeds (0–2) matched the single-task models. Because the split is drawn on the multi-task union, each endpoint is evaluated on its labelled test rows with the decision threshold tuned on its labelled validation rows; the comparison with the single-task models is therefore at the level of the evaluation protocol rather than identical test partitions.

### 4.3. Leakage-Resistant Evaluation Protocol

Following the evaluation principles of Section 3, all endpoints were evaluated with repeated scaffold-group splitting: five repeats (seeds 0–4) for the fingerprint models and three repeats (seeds 0–2) for the D-MPNN, each partitioning compounds by Bemis–Murcko scaffold into 80% training, 10% calibration, and 10% test sets, so that no scaffold appears in more than one partition. This scaffold-aware splitting provides a more realistic assessment of the model’s ability to generalize to novel chemical structures than random splitting, as it ensures that compounds with similar core scaffolds do not appear in both training and test sets. All data-dependent operations, i.e., model fitting, probability calibration, decision-threshold selection, and conformal calibration, were fitted strictly within the corresponding training/calibration partitions, ensuring unbiased performance estimates.

Discrimination was summarized by AUROC, AUPRC, Matthews correlation coefficient (MCC), balanced accuracy, sensitivity, and specificity, with the decision threshold chosen to maximize the F1 score on the calibration partition only (for the D-MPNN, the scaffold-balanced validation partition served as the calibration partition). Probabilities were post-hoc calibrated with isotonic regression fitted on the calibration partition, and calibration quality was measured by the Brier score and expected calibration error (ECE). The Brier score measures the mean squared error between predicted probabilities and binary outcomes, while ECE partitions predictions into equally sized confidence bins and computes the weighted average difference between accuracy and confidence within each bin.

Uncertainty was quantified with class-conditional (Mondrian) split-conformal prediction [27,37] at miscoverage level α=0.1: nonconformity scores $1-{\hat{p}}_{\mathrm{true}\;\mathrm{class}}$ were computed on the calibration partition separately for each class, and the per-class quantile defines the prediction sets. We report overall empirical coverage, per-class coverage, and mean prediction-set size on the test partition. This class-conditional approach ensures that coverage guarantees are maintained separately for the active and inactive classes, which is particularly important for imbalanced datasets where the active class is of primary interest but also the class with the smallest sample size.

### 4.4. Explainability and Prioritization Demonstration

Feature attributions were computed for the XGBoost models with TreeSHAP [38,39] on 2000 held-out compounds per repeat. To quantify whether explanations are reproducible, we computed the pairwise Jaccard overlap between the top-20 most important features (by mean |SHAP|) across the five independently trained models of each endpoint, and mapped the most important fingerprint bits back to example substructures for chemical interpretation. This stability analysis addresses a critical but rarely tested assumption in explainable AI: that attributions are reproducible across independently trained models. Low stability would indicate that explanations are sensitive to the specific data split and model initialization, raising concerns about their reliability for guiding compound optimization.

To demonstrate Layer 6, XGBoost models for *S. aureus* activity and human cytotoxicity were trained on an 80% scaffold-group split of each curated table (seed 42), with a held-out 10% calibration partition used for isotonic calibration and Mondrian conformal scoring, and used to score all 406,959 natural products. Three objectives were computed per candidate: (i) an uncertainty-adjusted activity confidence, defined as one minus the Mondrian conformal *p*-value of the candidate against the inactive class; (ii) an uncertainty-adjusted safety confidence, defined as the conformal *p*-value against the non-toxic class; and (iii) structural novelty, calculated using the ECFP/Tanimoto novelty score $N_{i}$ defined in the Structural Novelty and Diversity-Aware Candidate Selection subsection above [50]. The score ranges from 0 (identical to a training compound) to 1 (maximally dissimilar).

The three-dimensional Pareto front of (activity confidence, safety confidence, novelty) was computed with an exact skyline algorithm, and a diversity-aware top-*k* selection (k=100) was applied to the front by greedy Bemis–Murcko scaffold traversal in order of decreasing balanced score (the mean of the three objectives). Prioritization quality was quantified by enrichment of the 588 flagged known antibiotics among top-ranked candidates, excluding exact InChIKey matches to training compounds, together with the scaffold diversity of the shortlist. This demonstration illustrates the complete workflow from data through prediction, uncertainty quantification, and multi-objective prioritization to a diverse candidate shortlist.

The applicability-domain scores specified in Section 3.8 were computed for all 406,959 library compounds. The structural score equals one minus the novelty score defined above, i.e., the maximum ECFP Tanimoto similarity to any *S. aureus* training compound. For the embedding score, 300-dimensional learned representations (first feed-forward block of the trained single-task *S. aureus* D-MPNN, seed 0) were extracted for the library and training compounds; $d(z_{i},D_{train})$ was instantiated as the mean Euclidean distance to the k=5 nearest training compounds, and the temperature τ as the 95th percentile of the leave-one-out 5-nearest-neighbour distances within the training set (τ=0.155), so that exactly 95% of training compounds satisfy $AD^{emb}\geq e^{-1}$ (a one-sided 95% applicability domain). A compound is labelled inside the structural applicability domain when $AD^{struct}\geq 0.4$ and inside the embedding applicability domain when $AD^{emb}\geq e^{-1}$ (equivalently, d≤τ).

## 5. Results

The proof-of-concept evaluation of NP-GUARD was designed to assess the performance of the framework’s core components, i.e., predictive modelling, uncertainty quantification, explainability, and multi-objective prioritization, under a leakage-resistant scaffold-aware evaluation protocol that reflects the realistic challenge of discovering structurally novel compounds. Using four publicly available antimicrobial datasets spanning diverse data regimes, i.e., high-throughput primary screens, cytotoxicity assessments, and literature-curated bioactivity data, we evaluated Random Forest, XGBoost, and a directed message-passing neural network (D-MPNN) as baseline models of increasing complexity and representational capacity.

The results are organized into six thematic subsections that progressively build from foundational predictive performance to practical decision-support capabilities. First, we report predictive discrimination performance and calibration properties under scaffold splitting, establishing the baseline predictive capability of the models and quantifying the impact of dataset characteristics on evaluation stability. Second, we evaluate the pathogen-conditioned masked multi-task model specified in Section 3.7 against the single-task baselines. Third, we examine the stability of model explanations across independently trained models, addressing a critical but rarely tested assumption in explainable AI: that attributions are reproducible and therefore reliable for guiding compound optimization. Fourth, we demonstrate the uncertainty-aware prioritization of a large natural-product library, illustrating the end-to-end workflow from predictions to a diverse candidate shortlist and validating the multi-objective prioritization strategy. Fifth, we characterize the applicability domain of the screening library and of the prioritized shortlist. Finally, we synthesize the implications of these findings for antimicrobial discovery workflows, highlighting the practical utility of uncertainty quantification, diversity-aware selection, and the importance of rigorous evaluation protocols for translating computational predictions into experimentally actionable candidates.

### 5.1. Predictive Performance and Calibration

**Figure 2:**
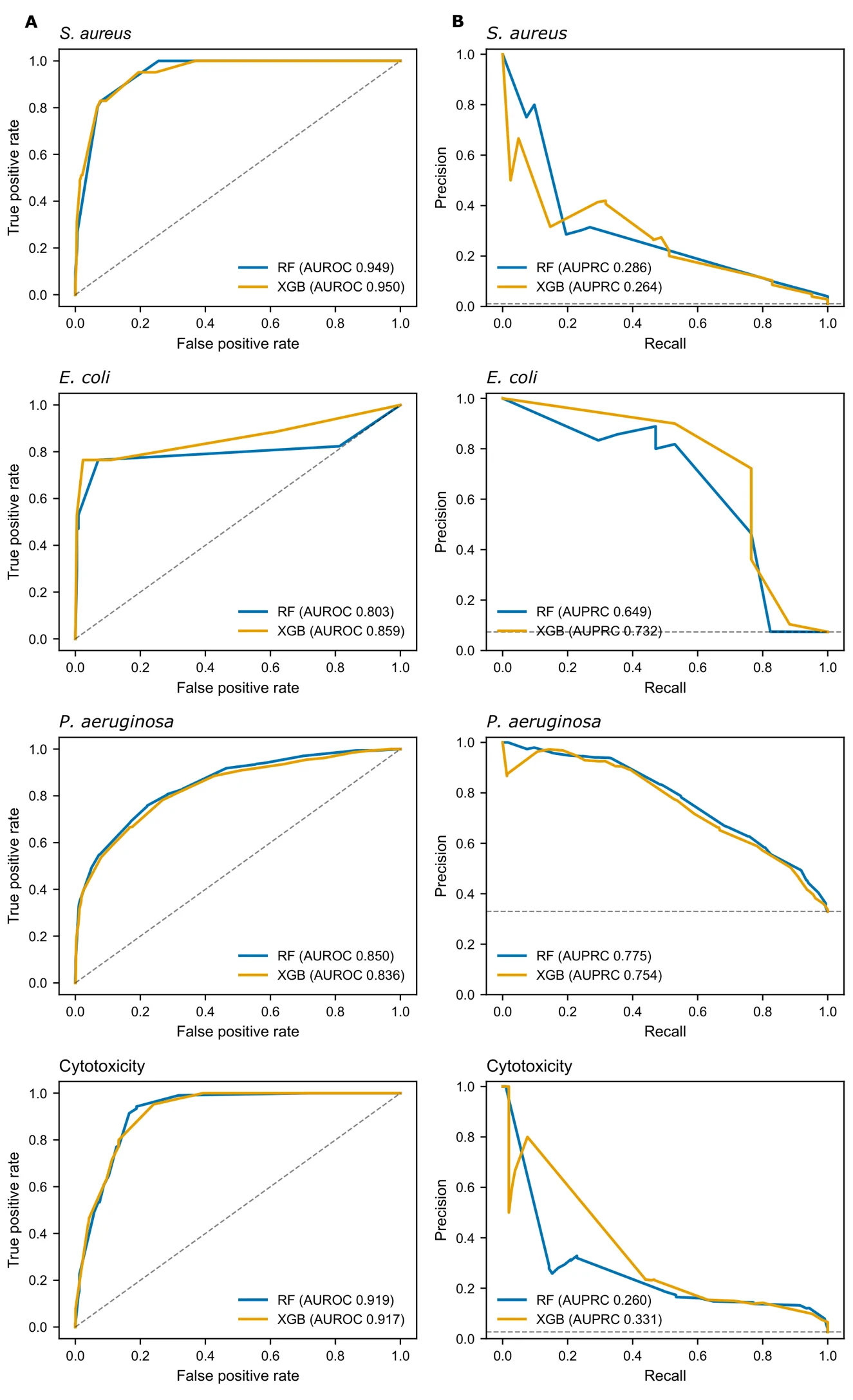
**Discrimination performance under scaffold-group splitting (repeat 0 shown)**. (**A**) Receiver-operating-characteristic (ROC) and (**B**) precision–recall curves for Random Forest and XGBoost on the four endpoints. Dashed lines indicate chance level (AUROC 0.5; AUPRC equal to the test prevalence). The ROC curves show strong discriminative performance across all endpoints, with AUROC values exceeding 0.9 for *S. aureus*, cytotoxicity, and *P. aeruginosa*. The PR curves reveal the practical implications of class imbalance, with substantially higher precision across all recall levels for the balanced *P. aeruginosa* endpoint compared to the highly imbalanced *S. aureus* and cytotoxicity endpoints.

Table 2 reports test performance across five repeated scaffold splits. Figure 2 provides a graphical overview of predictive discrimination across the four endpoints under the same scaffold-aware evaluation, complementing the numerical comparisons. Figure 2A displays the receiver-operating-characteristic curves, whereas Figure 2B presents the precision–recall curves, which make the effects of class imbalance on the retrieval of active compounds particularly apparent. Discrimination is strong for all endpoints despite scaffold separation: AUROC ranges from 0.85 to 0.93 for *S. aureus*, cytotoxicity and *P. aeruginosa*, and 0.78 to 0.82 for *E. coli*. However, performance on minority classes is modest and highly variable for imbalanced prospective screens: for *S. aureus*, AUPRC is 0.34 ± 0.09 (RF) and 0.29 ± 0.07 (XGBoost) against a 1.3% prevalence, and sensitivity at the calibration-tuned threshold is only 0.21–0.42. For *E. coli*, MCC varies from 0.40 ± 0.37 (RF) to 0.58 ± 0.18 (XGBoost), with individual repeats spanning nearly the full range. The literature-derived *P. aeruginosa* endpoint is the largest and most stable (AUROC 0.87 ± 0.01, AUPRC 0.79 ± 0.01, MCC 0.56 ± 0.02 for RF), illustrating how dataset size and class balance dominate evaluation variance. XGBoost improves sensitivity and MCC over RF at a small specificity cost, while RF attains slightly higher AUROC on all endpoints.

The D-MPNN reference model did not exceed the fingerprint models on the two large prospective screens (*S. aureus*: AUROC 0.90 ± 0.03 vs. 0.93 ± 0.03 for RF), though it attained the best AUPRC, MCC and sensitivity on cytotoxicity (0.34 ± 0.03, 0.37 ± 0.04 and 0.47 ± 0.05) and the best AUROC on *E. coli* (0.83 ± 0.08). The D-MPNN was also the least stable model: over three *E. coli* seeds, AUROC spanned 0.76–0.91, and MCC ranged from 0.07 to 0.38. Even on the largest and most balanced endpoint (*P. aeruginosa*, 29,986 compounds, 33% actives), the D-MPNN did not improve over fingerprint models on ranking or thresholded metrics (AUROC 0.82 ± 0.01 vs. 0.87 ± 0.01 for RF), though it attained the highest sensitivity (0.72 ± 0.11) at substantial specificity cost. At these dataset sizes, data partitioning dominates architectural differences, supporting the evaluation-first design of Section 3.

Isotonic calibration on the held-out calibration partition yielded low calibration error on test data: Brier 0.011 and ECE ≤ 0.005 for *S. aureus*; Brier 0.13–0.14 and ECE ≈ 0.04 for *P. aeruginosa*, reducing calibration error two- to five-fold relative to uncalibrated scores (raw ECE up to 0.069).

**Figure 3:**
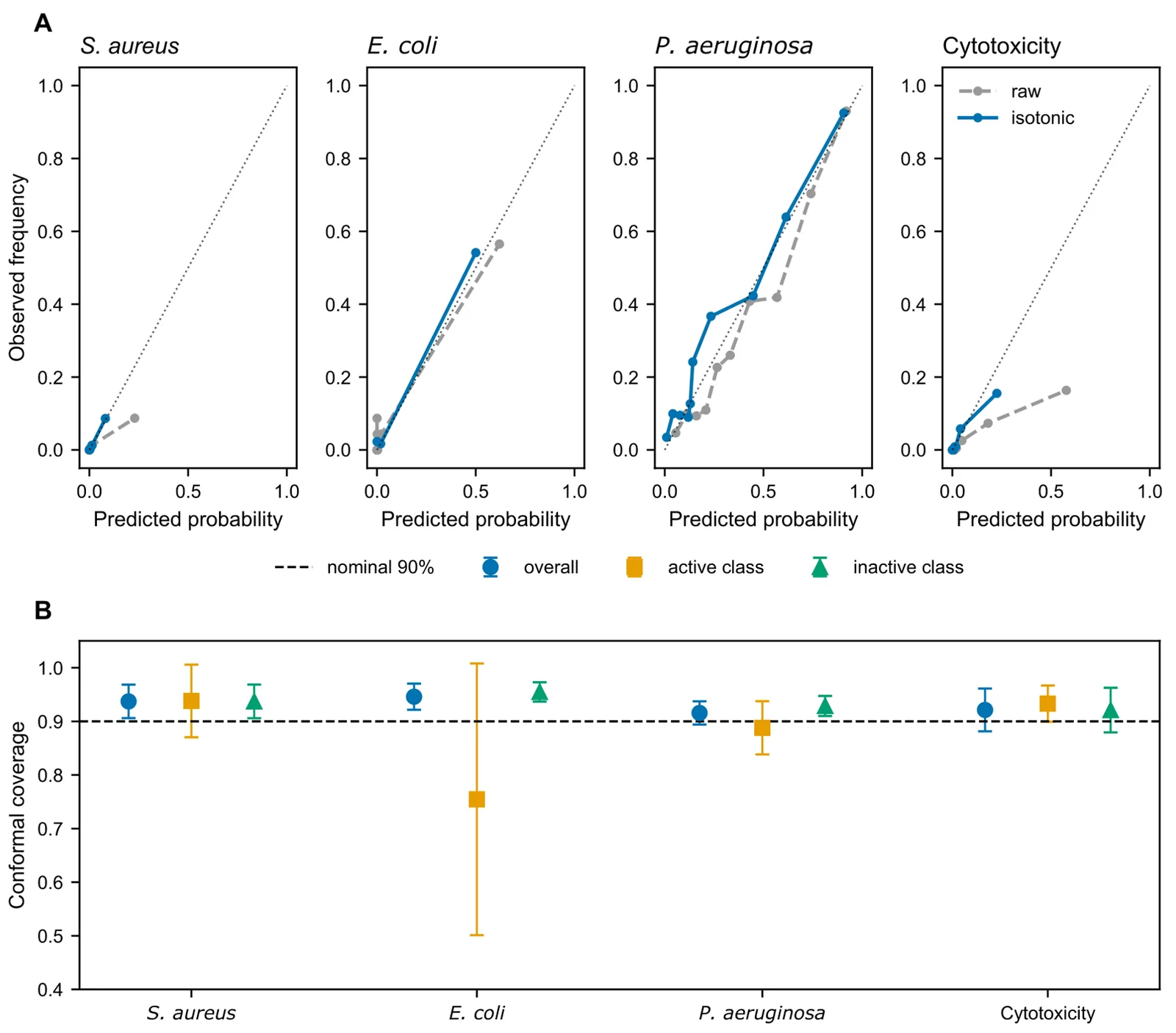
**Calibration and class-conditional conformal prediction (repeat 0, XGBoost)**. (**A**) Reliability curves before and after isotonic calibration on the held-out calibration partition. The dotted diagonal reference line indicates perfect calibration, with observed event frequency equal to predicted probability. Uncalibrated curves show systematic miscalibration across all endpoints; after isotonic calibration, the reliability curves align closely with the ideal diagonal, indicating effective probability calibration. (**B**) Empirical test coverage of Mondrian split-conformal prediction sets at nominal 90% coverage, overall and per class, across endpoints and models (mean ± SD over five repeats). Near-nominal overall coverage is achieved across all endpoints; per-class coverage remains close to nominal for both classes on the three larger endpoints, while *E. coli* shows reduced active-class coverage due to the limited number of calibration examples.

Figure 3 provides an integrated assessment of model probability calibration and class-conditional conformal coverage across the four endpoints, linking the quality of predicted probabilities with the reliability of the corresponding prediction sets under scaffold-aware evaluation.

Figure 3A presents reliability curves for XGBoost models before and after isotonic calibration on the held-out calibration partition. The uncalibrated curves show systematic miscalibration across all endpoints: predicted probabilities consistently overestimate observed frequencies for low-confidence predictions and underestimate them for high-confidence predictions. After isotonic calibration, the reliability curves align closely with the ideal diagonal, indicating that the calibration procedure effectively aligns predicted probabilities with observed outcomes. The calibration is particularly effective for *S. aureus* and cytotoxicity, where the calibrated curves nearly perfectly follow the diagonal, while for *P. aeruginosa* the calibration shows slight residual deviations in the mid-confidence range (0.4–0.6).

Table 3 summarizes the corresponding calibration and class-conditional conformal coverage metrics. Figure 3B reports the empirical test coverage of Mondrian split-conformal prediction sets at nominal 90% coverage, overall and per class, across endpoints and models (mean ± SD over five repeats). Mondrian split-conformal prediction at nominal 90% coverage achieved near-nominal empirical coverage overall (0.91–0.95 across endpoints). Crucially, per-class coverage remained close to nominal for both actives and inactives on the three larger endpoints (e.g., *S. aureus* XGBoost: 93.8% active-class vs. 93.7% inactive-class coverage). The error bars show the standard deviation across five scaffold splits, revealing that coverage stability depends primarily on dataset size: the largest and most balanced endpoint (*P. aeruginosa*) exhibits the narrowest error bars, while the small *E. coli* dataset shows the widest variability. For *E. coli*, where the calibration partition contains only ≈12 actives, active-class coverage was below nominal and highly variable (0.75 ± 0.16–0.25), quantifying the finite-sample limits of conformal calibration on very small minority classes. Mean prediction-set sizes (1.1–1.4 of two classes) confirm that conformal sets are informative rather than trivially large.

### 5.2. Pathogen-Conditioned Masked Multi-Task Prediction

Section 3.7 specifies a pathogen-conditioned masked multi-task predictor as the framework’s predictive layer. We implemented this layer as a single D-MPNN with four classification heads (*S. aureus*, *E. coli* and *P. aeruginosa* activity, and cytotoxicity), trained on the union of the four curated endpoint tables (68,818 unique compounds; 39,002 compounds labelled for at least two endpoints) with unobserved labels masked out of the training loss (Section 4.2). Table 4 compares the multi-task model against the single-task D-MPNN baselines under the same evaluation protocol.

Relative to the single-task baselines, multi-task training changes mean AUROC by +0.010/+0.025/+0.009/−0.011 and mean AUPRC by +0.007/+0.019/+0.015/−0.017 (*S. aureus*/*E. coli*/*P. aeruginosa*/cytotoxicity). The clearest benefit of multi-task training is expected for the data-scarcest endpoint, *E. coli* (2291 labelled rows), which can borrow statistical strength from the three larger endpoints through the shared message-passing encoder. This is borne out: *E. coli* AUPRC changes by +0.019 under multi-task training. Overall, the multi-task layer provides a single unified predictor for all four endpoints with the per-endpoint differences detailed in Table 4, and it is the architecture assumed by the framework specification; the single-task models are retained as the ablation baseline. Per-endpoint metrics for every seed are provided in the accompanying data release.

### 5.3. Explanation Stability Across Independently Trained Models

TreeSHAP attributions for the *S. aureus* XGBoost models ranked physicochemical descriptors (cLogP, molecular weight, QED) alongside fingerprint bits (e.g., bits 1905, 834 and 610, mapping to phenol-like and chiral aminoalkyl substructures) as dominant drivers of predicted activity. These attributions are chemically plausible: cLogP reflects lipophilicity influencing membrane penetration; molecular weight correlates with bioavailability; QED is a composite drug-likeness score; and the fingerprint bits correspond to structural features common in bioactive natural products.

**Figure 4:**
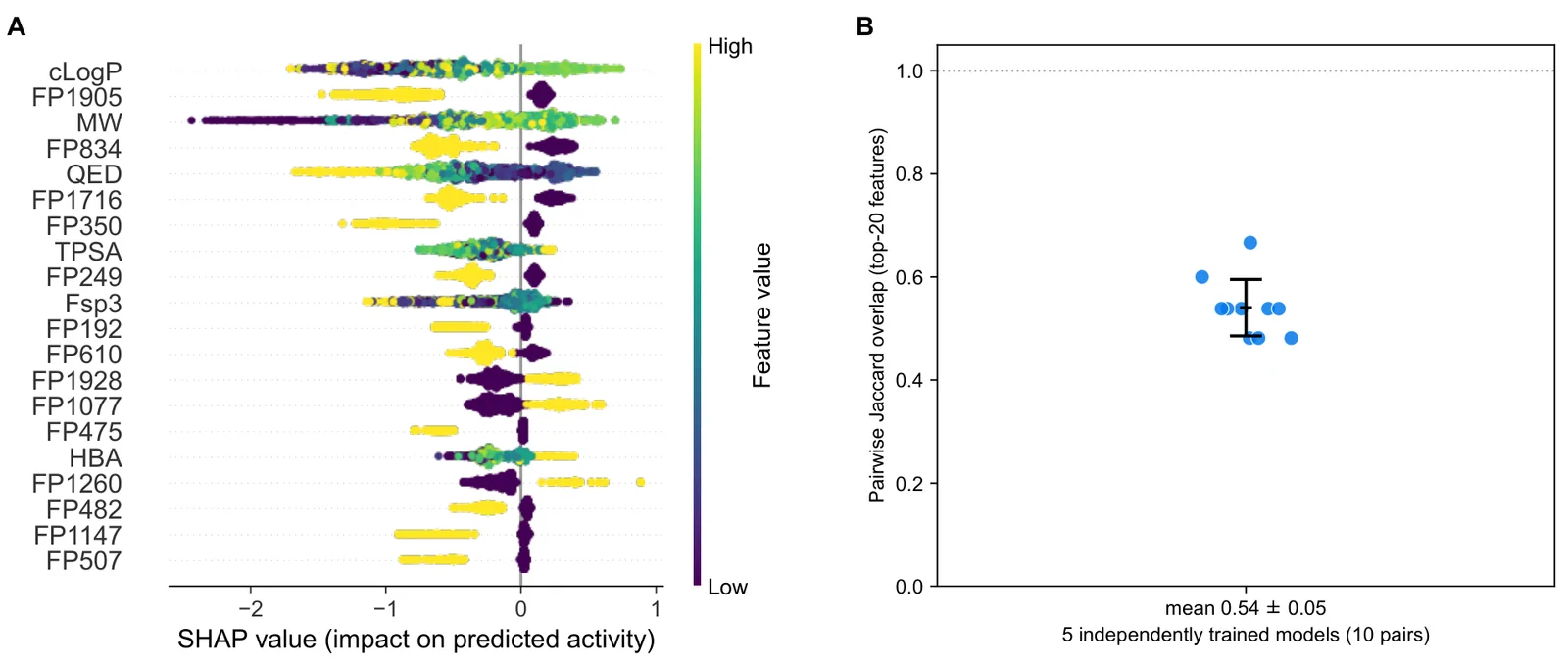
**Explainability of the** ***S. aureus*** **XGBoost models**. (**A**) TreeSHAP beeswarm of the 20 most important features (2000 held-out compounds; FP*n* denotes Morgan fingerprint bit *n*). The features are ordered by importance; cLogP and molecular weight show the largest absolute SHAP values, with high cLogP and low molecular weight positively associated with predicted activity. (**B**) Stability of top-20 feature sets across five independently trained models, measured by pairwise Jaccard overlap. Individual observations represent pairwise comparisons among the independently fitted models. The dotted horizontal line at a Jaccard similarity of 1.0 indicates perfect agreement between feature sets. The ten blue points correspond to the ten pairwise comparisons among five independently trained models, and the black summary mark indicates a mean overlap of 0.54 ± 0.05. This moderate agreement highlights the limited reproducibility of explanations.

Figure 4 presents the complementary interpretability analyses for the *S. aureus* XGBoost models, examining both which molecular descriptors and fingerprint features influence predictions and how consistently these important features recur across independently trained models. Together, the panels distinguish the interpretation of a fitted model from the reproducibility of that interpretation.

Figure 4A presents a TreeSHAP beeswarm plot of the 20 most important features for the *S. aureus* XGBoost model, based on 2000 held-out compounds. The beeswarm plot visualizes the distribution of SHAP values for each feature, with the figure’s purple-to-yellow color scale indicating lower to higher feature values, respectively. The features are ordered by importance, with cLogP, molecular weight (MW), and fingerprint bit 1905 (FP1905) showing the largest absolute SHAP values. For cLogP, higher values (yellow) are associated with positive SHAP values, indicating that increased lipophilicity drives predicted activity, consistent with the importance of membrane penetration for antibacterial activity. For molecular weight, lower values (purple) are associated with positive SHAP values, suggesting a preference for smaller, more bioavailable compounds. The fingerprint bits (FP1905, FP834, FP610) show mixed associations, with high values of FP1905 and FP834 positively associated with activity, while FP610 shows a more complex, non-linear pattern. The beeswarm plot reveals that feature effects are not strictly monotonic for many fingerprint bits; certain bit values contribute positively, others negatively, and the magnitude varies substantially depending on the molecular context, a characteristic of structural features that may be active only in specific chemical environments.

Figure 4B quantifies the stability of explanations across independently trained models. The pairwise Jaccard overlap of the top-20 attributed features across five independently trained models was only 0.54 ± 0.05. This means that approximately half of the top-ranked features change when the same model is refit on a different scaffold split of the same data. The scatter plot displays all ten pairwise Jaccard comparisons among the five fitted models as individual points, with a mean overlap of 0.54 ± 0.05 summarized by the black marker and error bar; the dotted line at 1.0 indicates perfect agreement. The observed points lie well below complete agreement, illustrating that feature rankings are only moderately reproducible. This level of instability is concerning for explanation-guided discovery: if the features highlighted as important vary substantially across different training runs, then explanations derived from a single model may not represent robust chemical insights. The instability likely reflects the high correlation among molecular features and the sensitivity of Shapley-based attributions to small changes in the model. Explanations of antimicrobial models should therefore be interpreted as hypotheses whose robustness must be quantified before guiding experimental design.

### 5.4. Uncertainty-Aware Prioritization of the COCONUT Library

As a Layer 6 proof-of-concept, we scored all 406,959 COCONUT natural products with *S. aureus* and cytotoxicity models, computing three objectives per candidate: activity confidence (one minus the Mondrian conformal *p*-value against the inactive class), safety confidence (conformal *p*-value against the non-toxic class), and structural novelty (1−max Tanimoto similarity to any *S. aureus* training compound).

**Figure 5:**
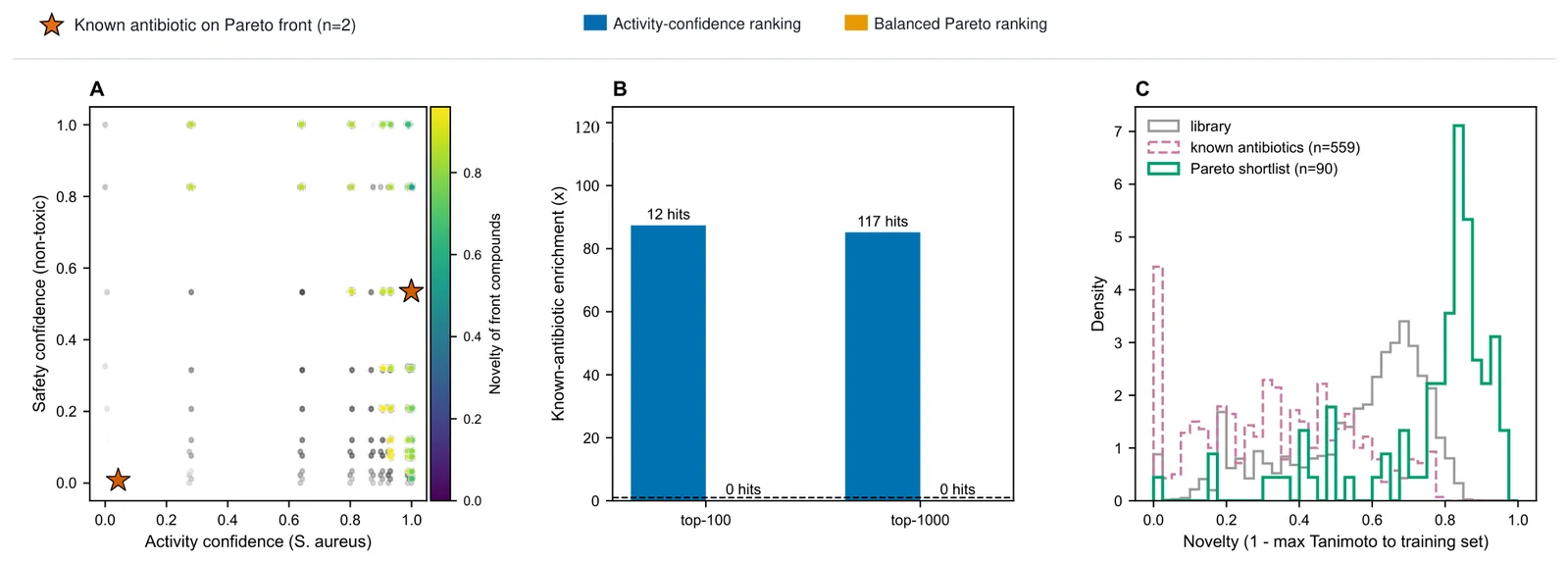
**Conformal-Pareto prioritization of the COCONUT natural-product library**. (**A**) Activity confidence versus safety confidence for the screening library (grey density), the 90-compound Pareto shortlist (colored by structural novelty on the purple-to-yellow scale), and the two known antibiotics on the front (orange stars). The Pareto front is positioned at the upper-right boundary of the distribution, representing optimal trade-offs between activity and safety. (**B**) Enrichment of known antibiotics under activity-confidence ranking versus the balanced multi-objective Pareto ranking, relative to random selection (dashed line); labels give absolute hit counts out of 559 evaluable known antibiotics. Activity-confidence ranking shows 87-fold enrichment in the top 100, while Pareto ranking deprioritizes known antibiotics in favor of novel chemotypes. (**C**) Distribution of structural novelty in the full library, among known antibiotics, and on the Pareto shortlist. The Pareto shortlist (mean novelty 0.76) is deliberately enriched for high-novelty compounds compared to the full library (mean novelty 0.61) and known antibiotics (median novelty 0.33).

Figure 5 summarizes the uncertainty-aware prioritization of the 406,959-compound COCONUT library by combining the activity–safety–novelty trade-off, retrospective enrichment of previously known antibiotics, and the structural-novelty distribution of the selected compounds. Taken together, these analyses distinguish the recognition of established active chemistry from the deliberate prioritization of chemically diverse, less familiar scaffolds.

Figure 5A visualizes the conformal-Pareto prioritization of the COCONUT natural-product library. Activity confidence is plotted against safety confidence for the screening library (grey density), with the 90-compound Pareto shortlist colored by structural novelty (purple-to-yellow scale, where yellow indicates higher novelty). The two known antibiotics on the front are marked with stars. The grey background points represent the displayed compound distribution from 406,959 candidates, with most compounds concentrated in the mid-range of both activity confidence (0.4–0.8) and safety confidence (0.3–0.7). The Pareto front is positioned at the upper-right boundary of this distribution, representing compounds that achieve the best trade-offs between high activity confidence and high safety confidence. The front’s spread across the novelty gradient (colored points) shows that the diversity-aware algorithm selects compounds spanning the full novelty range, with high-novelty compounds (red) interspersed with lower-novelty compounds (blue). The presence of two known antibiotics (stars) on the front, but their relatively low novelty scores, illustrates the trade-off between exploiting known active scaffolds and exploring novel chemical space.

Figure 5B presents the enrichment of known antibiotics under activity-confidence ranking versus the balanced multi-objective Pareto ranking, relative to random selection (dashed line). Ranking the library by activity confidence alone recovers 12 of 559 evaluable known antibiotics within the top 100 (87-fold enrichment; 117 within the top 1000, 85-fold). The activity-confidence ranking shows steep enrichment in the first few hundred candidates, recovering 413 of the 559 evaluable known antibiotics (74%) within the top 100,000 candidates. In contrast, the balanced multi-objective Pareto ranking recovers none of the known antibiotics in the top 100–1000 and only 102 (18%) within the top 100,000, because the novelty objective actively deprioritizes known compounds. This separation validates that the activity model recognizes validated chemistry (high enrichment when ranking by activity confidence alone) while the novelty objective—by design—deliberately deprioritizes rediscovery in favor of new chemotypes.

Two further analyses qualify the enrichment figure. First, because known-antibiotic flagging relies on deposited names, the flagged set is complete only within the named-compound stratum (356,602 compounds; the random-sample stratum contains no name-matched antibiotics by construction). Recomputing the activity-confidence enrichment within the named stratum alone yields 20 hits in the top 100 (128-fold; base rate 0.157%) and 140 in the top 1000 (89-fold): the library construction therefore does not inflate the reported enrichment, if anything, the full-library figure is conservative.

Second, to probe analogue-level leakage beyond exact InChIKey matches, we excluded all library compounds with Tanimoto similarity ≥0.4 to any *S. aureus* training compound. This filter removes 88% of the evaluable known antibiotics (65 of 559 remain), confirming that most known antibiotics are close analogues of training chemistry. On the remaining 214,528 compounds, activity-confidence enrichment drops to 0 hits in the top 100 and 2 in the top 1000 (6.6-fold over the residual base rate of 0.03%). The retrospective enrichment is thus dominated by rediscovery of training-like chemistry; the residual, analogue-free signal is statistically underpowered (0.3 hits expected per 1000) and is reported as a boundary condition rather than as evidence of de novo chemotype recognition. This is precisely the regime the novelty-seeking Pareto layer is designed for: the 90-compound shortlist contains no known antibiotics and has mean novelty 0.76.

Figure 5C shows the distribution of structural novelty in the full library, among known antibiotics, and on the Pareto shortlist. The full library (solid grey) has a broad distribution centered around 0.6, with a long tail extending to 1.0 (high novelty). Known antibiotics (pink dashed curve) are shifted toward lower novelty (median 0.33), reflecting their similarity to training compounds and their established chemical classes. The Pareto shortlist (green solid curve) is deliberately enriched for high-novelty compounds, with its distribution shifted toward higher novelty values (mean 0.76 vs. 0.61 library-wide) and a distinct peak around 0.7–0.9. The shortlist’s mean safety confidence was moderate (0.45), illustrating that the front surfaces compounds whose activity evidence is strong but whose cytotoxicity evidence is ambiguous, precisely the candidates for which experimental profiling is most informative. This uncertainty-aware prioritization ensures that experimental resources are directed toward compounds with the most reliable evidence of activity while still exploring novel chemical space.

### 5.5. Applicability Domain of the Screening Library

**Figure 6:**
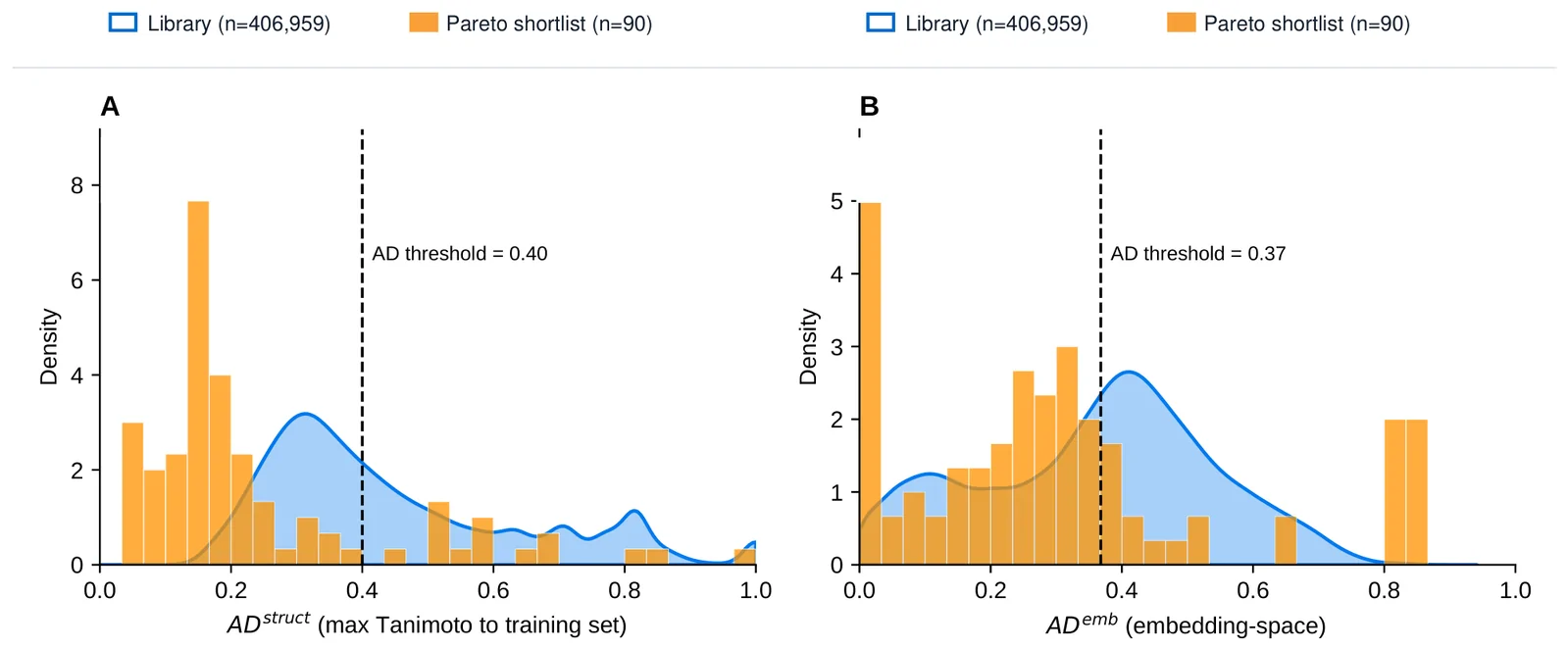
**Applicability-domain (AD) distributions for the screening library (blue density curves) and the 90-compound Pareto shortlist (orange histograms)**. (**A**) Structural AD score ($AD^{struct}$, maximum ECFP Tanimoto similarity to the *S. aureus* training set); the dashed line marks the in-domain threshold $AD^{struct}=0.4$. (**B**) Embedding AD score ($AD^{emb}=e^{-d/\tau }$, with *d* the mean distance to the five nearest training compounds in the D-MPNN representation space and τ the 95th percentile of leave-one-out training distances); the dashed line marks $AD^{emb}=e^{-1}$. The shortlist is deliberately shifted out of domain on both axes: 16.7% and 27.8% of shortlist compounds lie inside the structural and embedding ADs, versus 46.6% and 54.0% of the library.

Because the screening models are trained almost entirely on synthetic compounds and applied to natural products, we computed the two applicability-domain (AD) scores specified in Section 3.8 for all 406,959 library compounds (Section 4.4). Figure 6 compares the applicability-domain distributions of the complete screening library and the 90-compound Pareto shortlist, illustrating the consequences of novelty-oriented selection for the reliability of model extrapolation.

Figure 6A shows the structural similarity-based applicability domain, whereas Figure 6B shows applicability in the learned molecular embedding space; both panels indicate their respective in-domain thresholds. The structural score $AD^{struct}$ (maximum ECFP Tanimoto similarity to any *S. aureus* training compound) has median 0.39 (IQR 0.30–0.56) across the library, with 46.6% of compounds inside the structural AD ($AD^{struct}\geq 0.4$); the embedding score $AD^{emb}$ has median 0.38, with 54.0% of compounds inside the embedding AD (d≤τ). The two scores are only weakly correlated (Spearman ρ=−0.15), confirming that they capture complementary notions of proximity, i.e., structural resemblance to training chemistry versus proximity in the activity-trained representation space. Known antibiotics are strongly enriched inside the structural AD (88.9%; median $AD^{struct}$ 0.69) but not the embedding AD (28.1%), consistent with the analogue analysis above.

The 90-compound Pareto shortlist is deliberately shifted out of domain (Figure 6): median $AD^{struct}$ 0.17, with only 15 of 90 compounds (16.7%) inside the structural AD, and median $AD^{emb}$ 0.28, with 25 of 90 (27.8%) inside the embedding AD. This is the expected consequence of the novelty objective, and it sharpens how the shortlist should be read: the 15 in-domain compounds are the higher-confidence prioritized hypotheses, whereas the remaining 75 are out-of-domain candidates for which point predictions carry lower confidence and which therefore require greater experimental caution, precisely as Section 3.8 prescribes. Stratifying the retrospective enrichment by applicability domain shows that the activity-confidence ranking performs best inside the structural AD (9 hits in the top 100, 34.5-fold; 129 in the top 1000, 49.5-fold) and poorly outside it (0 and 2 hits, respectively), reinforcing that retrospective validation is informative mainly within the applicability domain, while prospective exploration outside it is the framework’s intended use.

## 6. Discussion

This study makes three principal contributions to the computational discovery of antimicrobial natural products. First, it specifies NP-GUARD, a modular architecture that reformulates antimicrobial natural-product discovery as an uncertainty-aware, relational, and multi-objective learning problem rather than as a single-classifier ranking task. Second, it provides a proof-of-concept evaluation of the framework’s data, predictive, uncertainty, explainability, and prioritization layers on public data for three priority pathogens and human cytotoxicity, using an evaluation protocol designed to measure discovery-relevant generalization. Third, it reports two empirical findings that we believe are broadly relevant to the antimicrobial machine-learning literature: the large split-to-split variance of minority-class metrics under scaffold separation, and the only moderate stability of feature attributions across independently trained models.

The separation of representation, prediction, trustworthiness, and decision-making into distinct, modular layers represents a significant departure from conventional virtual-screening pipelines. This separation ensures that the molecule receiving the highest predicted probability of activity is not automatically selected as the best experimental candidate. Instead, NP-GUARD explicitly represents and propagates multiple dimensions of evidence, i.e., antimicrobial activity, safety, novelty, uncertainty, and structural diversity, before making a recommendation. This design reflects the reality of antimicrobial drug discovery, where a compound must satisfy multiple, often competing, criteria to be a viable therapeutic lead. By integrating these dimensions into a unified framework, NP-GUARD provides a more principled basis for experimental prioritization than approaches that collapse all considerations into a single composite score.

The proof-of-concept evaluation reveals that scaffold-aware evaluation fundamentally changes the interpretation of model performance. Under repeated scaffold splitting, AUROC for the *S. aureus* endpoint varied between 0.89 and 0.97 across repeats, and *E. coli* MCC varied by more than 0.7 between splits of the same data. The effect was most extreme for the deep-learning baseline: across three *E. coli* scaffold splits, the D-MPNN’s AUROC spanned 0.76–0.91 and its MCC 0.07–0.38, so that the same model would appear either clearly superior or clearly inferior to the fingerprint baselines depending on the split chosen. Single-split evaluations, still common in the literature, can therefore substantially over- or under-state model quality, and differences smaller than this split variance should not be interpreted as evidence of model superiority. This observation directly motivates the repeated, leakage-resistant evaluation protocol specified in NP-GUARD’s design and underscores the importance of rigorous validation for any computational screening method that aims to discover genuinely novel chemotypes.

The class-conditional conformal analysis reveals a critical boundary condition for uncertainty-aware screening. For the prospective screens, sensitivity at an F1-tuned threshold remains below 0.5 even though AUROC exceeds 0.9: with ≈1% prevalence, ranking quality and calibrated decision-making matter more than threshold-free discrimination. The class-conditional conformal analysis shows that reliable per-class prediction sets are achievable on the larger endpoints, but that the smallest dataset (*E. coli*, ≈12 calibration actives) sits at the finite-sample limit of the method, an honest boundary condition for uncertainty-aware discovery on rare phenotypes. This finding highlights the importance of dataset size for conformal prediction: while the method provides finite-sample coverage guarantees in theory, these guarantees become unreliable when the number of calibration examples is extremely small. Practitioners applying conformal prediction to antimicrobial screening should therefore ensure that their calibration sets contain at least several dozen examples from each class.

The moderate top-feature overlap across refits (Jaccard 0.54) indicates that individual SHAP rankings should not be over-interpreted as stable mechanistic claims. The instability likely reflects the high correlation among molecular features, many fingerprint bits and physicochemical descriptors are collinear, and the sensitivity of Shapley-based attributions to small changes in the model. This finding challenges a common assumption in the application of explainable AI to drug discovery: that explanations derived from a single model provide reliable guidance for compound optimization. NP-GUARD therefore treats explanation stability as a first-class evaluation quantity, and we recommend that explanation-guided compound optimization be restricted to features that recur across resampled model fits. Only explanations demonstrating sufficient recurrence across independently fitted models should be treated as robust mechanistic hypotheses, converting XAI from a purely illustrative component into an experimentally assessable property of the computational framework.

The COCONUT prioritization demonstration separates two questions that are often conflated in virtual-screening studies: whether the predictive model recognizes validated chemistry and whether the prioritization layer proposes new chemistry. Ranking the library by activity confidence alone recovers 12 of 559 evaluable known antibiotics within the top 100 (87-fold enrichment), confirming that the predictive layer recognizes validated chemistry, although analogue-exclusion analysis shows that this retrospective signal is dominated by training-like chemistry: after removing compounds with Tanimoto ≥0.4 to the training set, too few known antibiotics remain (65) to power the enrichment test. However, the balanced multi-objective Pareto ranking yields a 90-compound shortlist spanning 72 unique scaffolds, with mean novelty 0.76 versus 0.61 library-wide, and only two known antibiotics on the front. By design, the novelty objective deprioritizes rediscovery in favour of new chemotypes. Reporting only the first number would overstate the practical novelty of the output; reporting only the second would leave the model unvalidated. We suggest that future virtual-screening studies report both quantities explicitly, distinguishing between the validation of the predictive model and the novelty of the proposed candidates.

The shortlist’s mean safety confidence was moderate (0.45), illustrating that the Pareto front surfaces compounds whose activity evidence is strong but whose cytotoxicity evidence is ambiguous, precisely the candidates for which experimental profiling is most informative. This is a deliberate feature of the conformal-Pareto prioritization strategy: by focusing on compounds with reliable activity evidence but uncertain safety profiles, the framework directs experimental resources toward the compounds where additional data would be most valuable for decision-making. This uncertainty-aware prioritization distinguishes NP-GUARD from conventional ranking approaches that treat uncertainty as an afterthought rather than integrating it into the decision-making process.

Several limitations qualify these results. The proof-of-concept uses fingerprint/gradient-boosting baselines and a standard D-MPNN rather than the full multi-view encoder and pathogen-conditioned mixture-of-experts model; the benchmark therefore quantifies the evaluation protocol and data foundation, not the ceiling of the architecture. The *P. aeruginosa* endpoint is derived from literature aggregation and its class balance reflects publication bias, limiting its utility as a prospective screening benchmark. The screens measure growth inhibition at a fixed concentration, so the labels conflate potency with exposure and assay artifacts. Cytotoxicity is represented by three cell lines at a single concentration and does not capture in vivo tolerability. The prioritization demonstration is retrospective; prospective experimental validation of ranked natural products is the decisive next step. Conformal coverage on very small minority classes is not guaranteed to be near nominal, as the *E. coli* results show.

Despite these limitations, the practical implications of this work extend beyond the specific application to antimicrobial natural-product discovery. The principles underlying NP-GUARD, i.e., uncertainty-aware multi-objective prioritization, explanation stability analysis, and leakage-resistant evaluation, are applicable to a wide range of molecular discovery problems, including drug repurposing, toxicity prediction, and materials discovery. The framework’s modular design enables the integration of additional data types, including protein–ligand interaction data, genomic information, and phenotypic screening data, as they become available. The provenance-aware knowledge base provides a foundation for reproducible research and collaborative model development, enabling researchers to share both data and models in a transparent, traceable manner.

Looking to the future, several directions for further development are particularly promising. The full deep architecture specified in Section 3, i.e., cross-modal graph-language encoding, natural-product domain adaptation, pathogen-conditioned multi-task learning, deep-ensemble uncertainty, and the provenance-aware knowledge base, will be implemented and benchmarked in subsequent work building on the curated data, protocol, and baselines reported here. The integration of predicted ADMET properties, including metabolic stability and permeability, into the multi-objective prioritization framework could further improve the selection of compounds with favourable pharmaceutical properties. The application of federated learning approaches could enable collaborative model training across multiple institutions without compromising data privacy, a particularly valuable capability for antimicrobial discovery where data sharing is often constrained by proprietary or regulatory considerations. Finally, prospective experimental validation of the ranked natural products is the decisive next step in establishing the practical utility of the NP-GUARD framework for antimicrobial natural-product discovery.

## 7. Conclusions

We presented NP-GUARD, a computational framework that reformulates antimicrobial natural-product discovery as an uncertainty-aware, relational, and multi-objective learning problem, and reported a proof-of-concept evaluation on public screening data for *S. aureus*, *E. coli*, and *P. aeruginosa* together with human cytotoxicity. The framework specifies six core components, i.e., provenance-tracked data curation, multi-view molecular representation, pathogen-conditioned multi-task prediction, explicit uncertainty quantification, stability-aware explainability, and conformal-Pareto prioritization, within a single, modular architecture that separates representation, prediction, trustworthiness, and decision-making. Of these, the present work implements and benchmarks the data, predictive, uncertainty, explainability and prioritization layers, including a first pathogen-conditioned masked multi-task graph model, while the multi-view encoder, domain adaptation and knowledge base remain specified for subsequent work.

Baseline models evaluated under repeated scaffold-aware splitting achieve strong discrimination but exhibit substantial split-to-split variance on minority-class metrics, demonstrating that scaffold-aware evaluation is essential for realistic assessment of model generalisation to novel chemotypes. Class-conditional conformal prediction provides near-nominal per-class coverage on adequately sized datasets, offering a principled mechanism for uncertainty-aware prioritisation; however, the finite-sample limits on very small minority classes underscore the importance of dataset size for reliable uncertainty estimation. Explanation stability across independently trained models is moderate, quantifying a critical caveat for explanation-guided discovery: only features that recur across resampled model fits should be treated as robust mechanistic hypotheses. Uncertainty-adjusted multi-objective ranking provides a principled route from predictions to experimentally actionable, diverse candidate shortlists, balancing activity, safety, and novelty while explicitly incorporating predictive uncertainty into the decision-making process.

The curated, provenance-tracked datasets, evaluation protocol, and baselines reported here constitute the reproducible foundation on which the full NP-GUARD architecture will be benchmarked. The framework is designed to transform conventional in silico screening from a ranking of molecular prediction scores into a transparent and reproducible system for deciding which natural products should be experimentally tested next, why they should be tested, and how confident the computational evidence supporting that decision actually is. By integrating representation learning, uncertainty quantification, explainability, and multi-objective optimization into a unified framework, NP-GUARD provides a comprehensive solution to the challenges of antimicrobial natural-product discovery in the era of deep learning and big data. The ultimate test of the framework will be prospective experimental validation of its predictions, a direction we are actively pursuing in ongoing work. As the global threat of antimicrobial resistance continues to escalate, computational frameworks like NP-GUARD offer a scalable, cost-effective complement to experimental screening, accelerating the discovery of the new antibiotics that the world urgently needs.

## Figures and Tables

**Table 1:** Summary of curated activity endpoints and the natural-product screening library used in the proof-of-concept evaluation. The four endpoints span complementary data regimes: high-throughput primary screens against *S. aureus* and *E. coli*; a parallel cytotoxicity screen against three human cell lines (HepG2, skeletal muscle, IMR-90 fibroblasts); and a literature-curated MIC dataset for *P. aeruginosa* from ChEMBL. Activity thresholds are defined as follows: growth inhibition ≥ 0.8 at 10 µM for *S. aureus*; binary label from the original study for *E. coli*; mean inhibition ≥ 0.5 across three human cell lines for cytotoxicity; and MIC ≤16 µg/mL for *P. aeruginosa*. The COCONUT screening library comprises 406,959 natural products, of which 588 known antibiotics are flagged exclusively for enrichment evaluation.

Endpoint	Source	Assay Type	Compounds	Actives (%)
*S. aureus* activity	Wong et al. [15]	HTS screen, 10 µM	38,995	497 (1.27)
Human cytotoxicity	Wong et al. [15]	3 cell lines, 10 µM	38,996	1024 (2.63)
*E. coli* activity	Stokes et al. [13]	growth screen	2291	119 (5.19)
*P. aeruginosa* activity	ChEMBL [44]	literature MIC	29,986	10,020 (33.42)
NP screening library	COCONUT [9]	—	406,959	588 ^a^

^a^ Compounds name-matched to known antibiotics; used only for enrichment evaluation, never for training.

**Table 2:** Test-set discrimination performance under repeated scaffold-group splitting (mean ± SD across five repeats for RF/XGBoost and three repeats for D-MPNN). Performance metrics include the area under the receiver operating characteristic curve (AUROC), the area under the precision–recall curve (AUPRC), the Matthews correlation coefficient (MCC), balanced accuracy, sensitivity, and specificity. Thresholds for classification were tuned exclusively on the held-out calibration partition to maximise the F1 score, ensuring that no test-set information influenced decision-threshold selection. Values in bold indicate the best performance per endpoint. The D-MPNN results are based on three repeats due to computational constraints.

Endpoint	Model	AUROC	AUPRC	MCC	Bal. Acc.	Sens.	Spec.
*S. aureus*	RF	**0.932 ± 0.028**	**0.341 ± 0.091**	0.291 ± 0.102	0.605 ± 0.044	0.214 ± 0.089	**0.996 ± 0.001**
	XGB	0.920 ± 0.026	0.291 ± 0.071	**0.359 ± 0.077**	**0.705 ± 0.050**	**0.422 ± 0.100**	0.989 ± 0.002
	D-MPNN	0.901 ± 0.028	0.287 ± 0.078	0.312 ± 0.037	0.699 ± 0.029	0.412 ± 0.060	0.986 ± 0.002
*E. coli*	RF	0.816 ± 0.061	**0.548 ± 0.189**	0.401 ± 0.370	0.657 ± 0.149	0.318 ± 0.301	**0.997 ± 0.003**
	XGB	0.778 ± 0.138	0.534 ± 0.229	**0.578 ± 0.182**	**0.728 ± 0.116**	0.463 ± 0.236	0.993 ± 0.005
	D-MPNN	**0.830 ± 0.076**	0.463 ± 0.140	0.243 ± 0.156	0.657 ± 0.133	**0.567 ± 0.334**	0.747 ± 0.324
*P. aeruginosa*	RF	**0.868 ± 0.012**	**0.793 ± 0.013**	**0.556 ± 0.023**	0.752 ± 0.012	0.577 ± 0.024	**0.927 ± 0.010**
	XGB	0.848 ± 0.007	0.771 ± 0.015	0.531 ± 0.030	**0.762 ± 0.014**	0.666 ± 0.035	0.859 ± 0.033
	D-MPNN	0.818 ± 0.013	0.722 ± 0.005	0.467 ± 0.039	0.737 ± 0.010	**0.717 ± 0.108**	0.758 ± 0.118
Cytotoxicity	RF	**0.928 ± 0.016**	0.290 ± 0.112	0.245 ± 0.123	0.588 ± 0.050	0.182 ± 0.101	**0.993 ± 0.002**
	XGB	0.916 ± 0.013	0.294 ± 0.089	0.320 ± 0.071	0.711 ± 0.041	0.454 ± 0.083	0.968 ± 0.010
	D-MPNN	0.910 ± 0.011	**0.339 ± 0.032**	**0.374 ± 0.042**	**0.718 ± 0.016**	**0.471 ± 0.050**	0.966 ± 0.020

**Table 3:** Calibration and class-conditional (Mondrian) split-conformal prediction results at nominal 90% coverage (mean ± SD over five repeats). Probabilities were post-hoc calibrated using isotonic regression fitted on the held-out calibration partition. The Brier score and expected calibration error (ECE) quantify calibration quality, with lower values indicating better alignment between predicted probabilities and observed outcomes. Empirical coverage represents the fraction of true labels contained in the conformal prediction sets, with separate coverage reported for active and inactive classes. Mean prediction-set sizes indicate the average number of classes returned by the conformal predictor (1–2 for binary classification), with values close to 1 indicating high-confidence predictions and values approaching 2 indicating uncertainty. The finite-sample limits of conformal calibration are evident for *E. coli*, where active-class coverage falls below the nominal 90% level due to the very small number of calibration examples (≈12 actives).

Endpoint	Model	Brier	ECE	Coverage	Cov. (Actives)	Cov. (Inactives)	Set Size
*S. aureus*	RF	0.011 ± 0.002	0.005 ± 0.002	0.928 ± 0.009	0.921 ± 0.069	0.929 ± 0.009	1.140 ± 0.131
	XGB	0.011 ± 0.003	0.004 ± 0.003	0.937 ± 0.031	0.938 ± 0.068	0.937 ± 0.031	1.334 ± 0.260
*E. coli*	RF	0.033 ± 0.006	0.023 ± 0.004	0.934 ± 0.050	0.745 ± 0.155	0.943 ± 0.051	1.388 ± 0.366
	XGB	0.032 ± 0.005	0.025 ± 0.009	0.946 ± 0.024	0.755 ± 0.253	0.955 ± 0.018	1.425 ± 0.398
*P. aeruginosa*	RF	0.131 ± 0.010	0.036 ± 0.005	0.923 ± 0.023	0.908 ± 0.026	0.930 ± 0.027	1.351 ± 0.087
	XGB	0.139 ± 0.009	0.039 ± 0.007	0.916 ± 0.022	0.888 ± 0.050	0.929 ± 0.019	1.374 ± 0.079
Cytotoxicity	RF	0.019 ± 0.003	0.008 ± 0.002	0.905 ± 0.037	0.941 ± 0.074	0.905 ± 0.039	1.172 ± 0.104
	XGB	0.019 ± 0.003	0.007 ± 0.003	0.921 ± 0.040	0.933 ± 0.034	0.921 ± 0.042	1.212 ± 0.028

**Table 4:** Single-task versus pathogen-conditioned masked multi-task D-MPNN (mean ± SD over seeds 0–2, scaffold-balanced 80/10/10 splits, validation-tuned decision thresholds). The multi-task model is a single network with four classification heads trained on the union of the four endpoint tables with unobserved labels masked; each endpoint is evaluated on its own labelled test rows. Labelled rows per endpoint are given in parentheses.

	AUROC		AUPRC		MCC	
Endpoint	Single	Multi	Single	Multi	Single	Multi
*S. aureus* (38,995)	0.901 ± 0.028	0.912 ± 0.032	0.287 ± 0.078	0.294 ± 0.078	0.312 ± 0.037	0.367 ± 0.126
*E. coli* (2291)	0.830 ± 0.076	0.855 ± 0.024	0.463 ± 0.140	0.481 ± 0.152	0.243 ± 0.156	0.363 ± 0.107
*P. aeruginosa* (29,986)	0.818 ± 0.013	0.827 ± 0.034	0.722 ± 0.005	0.737 ± 0.059	0.467 ± 0.039	0.495 ± 0.053
Cytotoxicity (38,996)	0.910 ± 0.011	0.899 ± 0.005	0.339 ± 0.032	0.322 ± 0.098	0.374 ± 0.042	0.319 ± 0.100

## Data Availability

All data used in this study are derived from publicly available resources: the *S. aureus* and cytotoxicity screens of Wong et al. [15], the *E. coli* screen of Stokes et al. [13], *P. aeruginosa* MIC data from ChEMBL [44], and natural-product structures from COCONUT [9]. Curated endpoint tables, the scored screening library, processing and analysis scripts, trained-model predictions, and analysis notebooks will be made publicly available upon publication. All reported results are reproducible from fixed seeds (benchmark repeats: seeds 0–4 for RF/XGBoost, 0–2 for the D-MPNN; prioritization demonstration: seed 42), pinned software versions (RDKit 2025.03.5 for the single-task analyses and 2026.3.6 for multi-task training and embedding extraction, scikit-learn 1.7.1, XGBoost 3.2.0, Chemprop 2.3.1), and the explicit activity thresholds of Table 1.
